# Supplementary material for: Barriers and facilitators to community acceptability of integrating point-of-care testing to screen for sickle cell disease in children in primary healthcare settings in rural Upper East Region of Northern Ghana
Source: PLoS One. 2024 May 20;19(5):e0303520. doi: 10.1371/journal.pone.0303520 (PMC11104616; doi:10.1371/journal.pone.0303520)
Supplement: S1 Data — (ZIP) [file pone.0303520.s001.zip › S1_Data for community members/A Awareness of SCD.docx]

**Name:** Awareness of SCD and name in local language

<Files\\FGDs\\FGD with under 5 mothers-Chiana Yidania-07> - § 6 references coded [5.99% Coverage]

Reference 1 - 1.75% Coverage

R2: Is true some children get sick frequently, it would be malaria or too much cold. That is why they get sick frequently.

Thank you.

I: Okay. Who has something to add?

R3: Thank you. The question you ask was do we know that there is something that causes children to get sick.

R3: As for me, I am not aware of that, I know of rainy season or when the child eventually is attack by sickness. So am not aware there is something that causes children to get sick.

Reference 2 - 0.60% Coverage

4: Thank you. It would be there but I have not seen it among the children in this community. Someone may have it but

R4: we do not know the name in Kasem. Thank you.

Reference 3 - 0.95% Coverage

R4: I have never heard of its name in kasem the usually describe it as sickle cell in the hospital. They mostly say they want to test you for sickle cell disease and when you are positive, your treatment is always different from those who do not have it.

Reference 4 - 0.81% Coverage

R3: Thank you. In this community we do not see it to be sickness in our bodies that is affecting us but looking at it critically, it is affecting us and in the community they do not regard it to be anything. Thank you.

Reference 5 - 0.81% Coverage

R3: If you do not have the sickle cell disease, you are able to fight disease quickly comparing to someone who has the disease. The one who has the sickle cell disease gets down to every sickness that attacks him/her.

Reference 6 - 1.07% Coverage

R5: when it comes to the children, they are weak with no strength and they can’t breastfeed well.

R4: when it comes to the children, they can’t eat and they are very weak as well and if it’s those who can’t even talk, then the case becomes worse so that’s how we know it is sickle cell.

<Files\\FGDs\\FGD with under 5 mothers-Mirirgu-06> - § 5 references coded [3.65% Coverage]

Reference 1 - 1.05% Coverage

R9: We have a disease like that example is rushes. You can give birth to a child and the child will be experiencing skin rushes once in a while. You will send the child to the hospital and they will treat it and it will be better for some days and come again. So, it is just in the child’s body; anytime it can just start developing in the child’s body.

Reference 2 - 0.91% Coverage

R2: One of my sisters gave birth to a child and the child died. The child didn’t even experience a good health condition for the whole of his life. The child was just falling sick every day like that. The child’s veins were seen out and also a bloated stomach. So, it didn’t keep long before the child died.

Reference 3 - 0.28% Coverage

M: What is the local name or English name for that disease?

R9: They always say it is HIV/AIDS.

Reference 4 - 0.95% Coverage

R8: Me too I have seen it, it is a regular heartbeat. Parents can also transfer a regular heartbeat to their child if the parents are having a regular heartbeat. It can also be asthma. One was having it and every three months they will send the child to the hospital and give him oxygen before he can be able to breathe.

Reference 5 - 0.46% Coverage

R2: The local name of sickle cell disease is called Ziim Poe baase (a disease in the blood)

R4: The local name is Koba Puan baase (a disease in the bones)

<Files\\FGDs\\FGD-Opinion Leaders- Chaina Assunia-04> - § 3 references coded [1.62% Coverage]

Reference 1 - 0.49% Coverage

R3: we see it like that in our communities because we see some children who fall sick always since infancy, so we have seen some.

Reference 2 - 0.67% Coverage

I: Okay, thank you. Please the disease I mentioned as sickle cell, do you have a local name for it in the community?

R2: in our community, we call suck sick children as sicklers.

Reference 3 - 0.46% Coverage

R4: as for this sickness, we do not know the exact name in Kasem but we refer to it as sickler just like my brother said.

<Files\\FGDs\\FGD-opinion leaders -Mirigu-05> - § 6 references coded [4.44% Coverage]

Reference 1 - 0.62% Coverage

R4: Yes, it is true T they give birth to children that normally fall sick easily. Every time they keep sending them to the hospital for treatment but their conditions don’t change for the better. We don’t also know what kind of disease is in the child so, they give birth to such children in this our community.

Reference 2 - 1.00% Coverage

R2: Since they gave birth to us, we keep seeing people giving birth to children and their bodies are always weak and tiny. And every day the parents keep going everywhere looking for herbs to give to the child but all their efforts don’t always better the child’s condition. So, most people call that condition or disease Puah (Malaria). Sometimes, the parents will manage and it becomes normal but within a short period, it will develop again in the child so, in the end, some children normally died.

Reference 3 - 0.23% Coverage

M: What is the name of sickle cell in the local language?

R7: We call it Ziim poe baansi (a disease in the blood)

Reference 4 - 0.83% Coverage

R9: It is a big thing for us to get you the actual local name for the disease. We always look at the signs and symptoms of the disease and give it a name. But because this disease normally makes your entire body weak and tiny, we called it Agula baansi (weak body disease).

Every day you are lying down weak. So,

if you go and marry a woman and she is having Agula blood, then you will give birth to an Agula child.

Reference 5 - 0.82% Coverage

R2: For the truth, this sickle cell disease doesn’t have actual name in our language.

Technology has come so in the hospitals; they can take your blood samples test and see that the blood has turned into a sickle shape and they now gave the name sickle cell. But we cannot see anything, we only see the appearance of the child’s condition and give a name to it. So sometimes, we don’t have a specific name for it.

Reference 6 - 0.95% Coverage

R8: The conversation is good, but what I want to also add is that; we learned that sickle cell is a disease that is in the blood but not a disease that is caused by the consumption of maggie food. So, unless you go and have a test in the hospital before you will know that you have the disease or you don’t have it. So,

it is good for you to test and know your sickle cell status so that you will be able to know what kind of food and medicine you will be taking to manage it.

<Files\\FGDs\\FGD-Opinion leaders-Chiana Saboro-08> - § 5 references coded [5.18% Coverage]

Reference 1 - 1.58% Coverage

7: We see people like that in the community the easily fall sick. I ever took someone to the hospital for blood transfusion, on my way back home the and told me that same person is in short of blood again and he was been given blood again and the next day the same thing happened again. We have seen many of such people in the community. So, we are happy you are coming to help people in such conditions.

Reference 2 - 0.50% Coverage

4: It is described as maleria in ancient times, the person loses weight and watery stool, this is what we know about the disease.

Reference 3 - 1.72% Coverage

9: The disease you describe as sickle cell, there are so many diseases in the olden days that they call bayapoga. When a person is suffering from this disease, the body peels off, swollen and losses blood gradually.

there is another one called bicharifufuga, this is a heart disease that was once there and causes children to lose weight and become weak.

This are all sickness in the olden days, we do not know the medicine to this sickness.

Reference 4 - 0.45% Coverage

9: Is a bad disease in this community, it causes us to lose their children and many people. This what I have to say.

Reference 5 - 0.93% Coverage

1: We don’t know the name but if it is about luck of blood among children it is common in Chaina. We do not know the name so, since you people know the name try and help us so that when we get the name of the disease, we will let you know.

<Files\\FGDs\\FGD-Opinion Leaders-Nabango-02> - § 5 references coded [1.98% Coverage]

Reference 1 - 0.47% Coverage

R9: Actually, I don’t know whether that thing they call Niila (Bird/ convulsion) or a different thing. This Niila disease also gets children at an early age.

Reference 2 - 0.14% Coverage

They called it Bayapeiliga (white undertaker).

Reference 3 - 0.34% Coverage

R3: We also know some people inside their bones, it is always paining them. And we called it Enduuma (body pains) FGD-Opinion Leaders-Nabango-02

Reference 4 - 0.58% Coverage

R4: The sickle cell patient is always anemic and also suffers from anemia. The sickle cell child’s color normally changes to white and that is why they called it Bayapeiliga (white undertaker).

Reference 5 - 0.45% Coverage

R9: Others also called it Tikanleipsi; (Abiku) the act of giving birth to children and they die and come back. They called such a child a Tikanlemiga.

<Files\\FGDs\\FGD-with under 5 mothers-Nabango-03> - § 2 references coded [0.74% Coverage]

Reference 1 - 0.23% Coverage

R9: Koba puan ba’asi is the local name (sickness in the bones)

Reference 2 - 0.52% Coverage

R8: This sickness in the bones normally makes the child to be very weak and cannot even wakeup.

<Files\\IDIs with SCD parents\\IDI-Parent with SCD patient-Doba-01> - § 3 references coded [2.55% Coverage]

Reference 1 - 0.78% Coverage

R: They said it is cold so the child is not supposed to expose to cold places and the child should not be bathing in cold water. That is what hospital doctors and nurses said but for my community Doba, I don’t know.

Reference 2 - 1.24% Coverage

R: They said it is demonian or something like that sort and the child is not supposed to expose to cold places or step his legs on cold places and he should always be at hot or a place with heat and should be bathing hot water. The child should be drinking plenty of water at the time. It was later that they said it was sickle cell disease.

Reference 3 - 0.53% Coverage

R: In my community, they normally said the sickness is bones sickness and it goes with the blood of the parents that is why some families are sickle cell patients.

<Files\\IDIs with SCD parents\\IDI-Parent with SCD Patient-Kologo-03> - § 3 references coded [1.13% Coverage]

Reference 1 - 0.37% Coverage

M: Are you aware some children are born with something in their bodies that makes them fall sick easily?

R: Yes.

Reference 2 - 0.51% Coverage

M: Okay, what is that something in their bodies that makes them fall sick easily?

R: It is a disease that is attacking my child, which is sickle cell disease. (IDI-Parent with SCD Patient-Kologo-03)

Reference 3 - 0.25% Coverage

M: How do they call it in your language?

R: Bone disease {koba puan baase}.

<Files\\IDIs with SCD parents\\IDI-Parent with SCD Patient-Korania-07> - § 1 reference coded [0.38% Coverage]

Reference 1 - 0.38% Coverage

I: what is the name in Kasem?

R: I do not know.

<Files\\IDIs with SCD parents\\IDI-Parent with SCD patient-Navrongo-02> - § 1 reference coded [0.57% Coverage]

Reference 1 - 0.57% Coverage

M: What did you people call sickle cell disease in your local language.

R: Please I don’t know what they called it in Kasem.

<Files\\IDIs with SCD parents\\IDI-Parent with SCD Patient-Nawognia-06> - § 3 references coded [2.34% Coverage]

Reference 1 - 0.36% Coverage

I: What do you know about it?

R: I do not know anything about it.

Reference 2 - 1.19% Coverage

I: But you are aware there are children who get sick frequently from birth?

R: Yes, I am aware some children are born with a disease and mostly admitted into the hospital and discharged and still get sick frequently.

Reference 3 - 0.80% Coverage

I: Do you know this disease name in Kasem or how do they call that disease that makes children fall sick frequently?

R: I have forgotten the name.

<Files\\IDIs with SCD parents\\IDI-Parent with SCD Patient-Paga-05> - § 2 references coded [2.69% Coverage]

Reference 1 - 1.30% Coverage

I: Please have you heard of a sickness that affects children and makes them fall sick more often?

R: Yes, I have, and even my rival has a child who is suffering from the same ailment. It is just recently that it is better with the child.

Reference 2 - 1.39% Coverage

I: What do they call it in the local language?

R: What I know is that, in our local language they call it ‘‘monkey’’ but the Ashanties also call it ‘assram’.

It makes the child will be dehydrated and the head alone will be noticed when you see the child.

<Files\\IDIs with SCD parents\\IDI-Parent with SCD-Pungu-04> - § 2 references coded [1.92% Coverage]

Reference 1 - 1.44% Coverage

R: When the child has the disease, the child can feel pains in the body especially in the bones. (IDI-Parent with SCD-Pungu-04)

, some children are even born with disease called ‘WEYOZUGA. This can be a disturbance to the child.

I: Do you know the name of this disease in the local language?

R: It is called ‘kachirikawiisa’ (bone disease).

Reference 2 - 0.48% Coverage

I: Kachirikawiisa? What is that?

R: It is a bone disease, it causes pains in the bones.
