# Supplementary material for: Barriers and facilitators to community acceptability of integrating point-of-care testing to screen for sickle cell disease in children in primary healthcare settings in rural Upper East Region of Northern Ghana
Source: PLoS One. 2024 May 20;19(5):e0303520. doi: 10.1371/journal.pone.0303520 (PMC11104616; doi:10.1371/journal.pone.0303520)
Supplement: S1 Data — (ZIP) [file pone.0303520.s001.zip › S1_Data for community members/B Perception of SCD.docx]

**Name:** Perception of families with SCD

<Files\\FGDs\\FGD with under 5 mothers-Chiana-01> - § 3 references coded [2.01% Coverage]

Reference 1 - 0.92% Coverage

R8: To me we in this community see this sickness to be a very bad one because you can lose someone you love at any point in time and that is very disturbing, so I think it is a bad sickness, that’s what I think. This is because you can lose you loved one. (FGD with under 5 mothers-Chiana-01)

Reference 2 - 0.31% Coverage

R8: I know but those persons are no more alive.

I: How many people?

R8: two people.

Reference 3 - 0.79% Coverage

R6: thank you. I see it to be normal because my brother’s wife has the sickness though my brother does not have it. When he was told, he took it normal and is still with the woman as we speak, so I see it to be normal.

<Files\\FGDs\\FGD with under 5 mothers-Mirirgu-06> - § 6 references coded [2.92% Coverage]

Reference 1 - 0.66% Coverage

R1: Some people normally think that it is a curse given to that family. Others will also think that the woman has gone against traditional rules which they called Kabunsi (A disease the woman gets when she commits adultery).

Reference 2 - 0.27% Coverage

R10: Other people will also be thinking that you don’t give proper health care to the child.

R8: Some people will also say you don’t give the child proper food to eat.

R3: People will also be saying you don’t practice personal hygiene which is why the child is affected by that disease.

Reference 4 - 0.44% Coverage

R2: Here in our community, people normally think that the family swears with the gods on something that is why the family members are sick that way.

R8: Other people also say those family members couldn’t fulfill the promise they made with the gods that’s why the family members are sick.

Reference 5 - 0.60% Coverage

R3: People always say that there is a disease in that family.

Reference 6 - 0.37% Coverage

R4: People also think that those people with that type of disease, are people that have used juju medicine on them that’s why.

<Files\\FGDs\\FGD-Opinion Leaders- Chaina Assunia-04> - § 1 reference coded [0.65% Coverage]

Reference 1 - 0.65% Coverage

R7: we see them as sicklers because they fall sick always and cannot do any meaningful work.

to be sustained. If they are sick, they become weak and this is how we view them.

<Files\\FGDs\\FGD-opinion leaders -Mirigu-05> - § 3 references coded [3.88% Coverage]

Reference 1 - 1.76% Coverage

R9: People will now be thinking badly about that family or that person who has it.

They normally think that family is a bad family, thinking that someone in that family wants to let the child suffer before killing the child.

R8: In a family with such a case, some people have fears to go and marry a man from such a family thinking that maybe one day the husband will die and leave her alone. So, fear to marry that family is always there.

R2: Other people usually think that the family members or the parents of the child made a promise to the gods and failed to fulfill the promise that is why the gods are now fighting them.

R2: Others will say the family members are witches, they want to cast a spell on the child before they can be able to kill the child that is why the child falls sick every day. So, people normally say a lot to a family with sickle cell disease.

Reference 2 - 0.94% Coverage

R6: I will also add something small. If some people always see a case like this in a family, they will be giving bad names to that family.

Even sometimes if they see that family child somewhere, they will be beating the child, giving the child abusive words.

So, it creates fears for people to be going to that family for friends. And sometimes, that family normally looks like it is not part of the community because the community People are discriminating against them.

Reference 3 - 1.17% Coverage

R7: People give bad names to families with sickle cell disease. If there is an old woman or old man in the family, they start to say that is those old women and the old man that are trying to kill the child or is the child’s mother or the father that has done something wrong somewhere that is why the child is suffering from that condition.

R9: People normally think that family or that person with sickle cell disease is a bad family or person. So even in school, friends don’t like playing with such people. Every public gathering, people have been disassociating themselves from them.

<Files\\FGDs\\FGD-Opinion Leaders-Nabango-02> - § 4 references coded [2.04% Coverage]

Reference 1 - 0.45% Coverage

R8: We normally think that child is a witch because they don’t always look like a living being.

Reference 2 - 0.79% Coverage

M: How do people who know your family has the disease see you?

R7: If a family is having that disease and whenever the family member gives birth and the child dies,

R7: People normally say that family members are witches that is why their children are dying like that.

Reference 3 - 0.56% Coverage

R4: Others will also be saying that their grandparents who are no more alive did something wrong with the gods during the time they were alive that is why the children are sick that way.

Reference 4 - 0.25% Coverage

R3: People will also be saying that family people are people that fall sick easily

<Files\\FGDs\\FGD-with under 5 mothers-Nabango-03> - § 3 references coded [2.71% Coverage]

Reference 1 - 0.83% Coverage

R4: Sometimes they will put the blame on the mother that she is the cause of that. Some will be saying the mother got the pregnancy from outside the family so the child is not part of the family that is why the child is like that

Reference 2 - 1.26% Coverage

M: How do people perceive families that have the disease?

R6: People normally think that a family is a sinful family that is why God is paying their debts.

R9: They always say they are wicked people

R3: They say that family is having a disease or that family is a Sickler

R8: Some say you like insulting people that is why you got that disease

Reference 3 - 0.61% Coverage

R10: Other people normally say if you are pregnant in some homes, you don’t go outside too much. If you are pregnant and you like going outside, you can get that disease

<Files\\IDIs with SCD parents\\IDI-Parent with SCD patient-Doba-01> - § 2 references coded [1.88% Coverage]

Reference 1 - 0.76% Coverage

M: Good, how do people perceive families that have the disease?

R: They normally said the sickness is bones sickness and it goes with the blood of the parents that is why some families are sickle cell patients

Reference 2 - 1.12% Coverage

M: Good but I am asking you how do people perceive families that have the disease?

R: That is what I already told you that people are saying that it is a bone sickness where people get it from the blood genes of their parents.

They normally classify those families as sick families or they are cursed by God.

<Files\\IDIs with SCD parents\\IDI-Parent with SCD patient-Navrongo-02> - § 2 references coded [2.40% Coverage]

Reference 1 - 1.46% Coverage

M: Excellent, how do people perceive families that have sickle cell disease?

R: They think it is spiritual and even to me, I think it is a spiritual thing in the families.

M: Please and what else again?

R: I won’t be there when they are discussing issues concerning that so I don’t know, please.

Reference 2 - 0.94% Coverage

M: Good, how do people who know your family has the disease think about you?

R: I don’t know what they will think about us and even no one knows that our child is suffering from that disease.

<Files\\IDIs with SCD parents\\IDI-Parent with SCD Patient-Nawognia-06> - § 2 references coded [3.45% Coverage]

Reference 1 - 2.18% Coverage

I: How do people see your family concerning Rostein who has sickle cell disease?

R: Some people see but they do not say anything,

others are always doubting and asking us to take him to the hospital to find out what exactly is wrong with the child.

him because anytime he gets an attack, he feels pains around his legs and hands meanwhile he did not fall, so people always say we should take him to the hospital

Reference 2 - 1.27% Coverage

R: What do people think about your family concerning Rostein’s condition? What do they say about your family?

R: Even when they discuss, you will not hear. They may talk among themselves but they never said something for me to hear.
