# Supplementary material for: Barriers and facilitators to community acceptability of integrating point-of-care testing to screen for sickle cell disease in children in primary healthcare settings in rural Upper East Region of Northern Ghana
Source: PLoS One. 2024 May 20;19(5):e0303520. doi: 10.1371/journal.pone.0303520 (PMC11104616; doi:10.1371/journal.pone.0303520)
Supplement: S1 Data — (ZIP) [file pone.0303520.s001.zip › S1_Data for community members/C Views on screening exercise at CHPS and clinics.docx]

**Name:** Views on screening exercise at CHPS and clinics

<Files\\FGDs\\FGD with under 5 mothers-Chiana Yidania-07> - § 3 references coded [5.16% Coverage]

Reference 1 - 1.83% Coverage

R2: We are really grateful for this kind gesture you are to do for us. We really appreciate, and this will help us test our children and know what is wrong with them and with your help we will find solutions to this disease.

before it is too late.

R3: some of us have our children and we don’t even know whether they have the sickle cell disease or not, so when you bring this machine, it will really help us know the status of our children.

Reference 2 - 2.33% Coverage

R4: we are happy and at the same time grateful for this machine because we are really suffering with our children, so this will really help us a lot. Thank you

R6: so when the machine comes, it will show whether or not the child has the sickness. So, I am really happy.

R7: as for me, I am really happy that this machine is coming and the fact that it is coming to help us is a cause for me to be happy.

R8: thank you for the machine, it will really help because when our children are sick a little it really disturbs us a lot. So, I am really happy we will get to know what is wrong with our children and get a cure for it.

Reference 3 - 1.00% Coverage

R9: I want to say thank you for the machine you are bringing to help us, when it comes and we test the children and we know what is wrong with them, it will help us know what to do and where to go so.

R5: as for me, I see it as a blessing and a help to us.

<Files\\FGDs\\FGD with under 5 mothers-Chiana-01> - § 4 references coded [3.46% Coverage]

Reference 1 - 0.84% Coverage

R6: I think it will help us a lot because it will help us test and know what is wrong with the children. So, when it is brought here and the children are tested positive, it will help us a lot and our children will be treated.

Reference 2 - 0.99% Coverage

R8: I think when you bring it, we will be happy because the children do not talk as we are holding the child, he/she cannot talk so when we test and know what is wrong with them, it will help. It is better to be aware than to be naïve and in want when the sickness strikes.

Reference 3 - 0.69% Coverage

R8: I am already happy because I see it as something that will bring a lot of help to most of us. This assistance will help us and I am already very happy even before the program will start.

Reference 4 - 0.95% Coverage

R9: what I also have to say is that this assistance coming to us has made me happy already as the nurses are here with us and they will take good care of us. So, if we all agree to accept it, it will be good for us and it will bring good health to our children.

<Files\\FGDs\\FGD with under 5 mothers-Mirirgu-06> - § 2 references coded [2.09% Coverage]

Reference 1 - 1.30% Coverage

M: What do you think about us doing the screening at the CHPs/Clinics?

R3: To me, it is good to come here and do it for us.

R5: It will be good because it will help all Mirigu people.

R3: If you bring the machine here for the testing, it will be good. Because we will now be able to know whether the children are having it or not.

R3: When they finish with the testing, they will also help us to know how to keep ourselves from getting it.

Reference 2 - 0.79% Coverage

R3: To me, it is a good thing if they bring the machine here for the testing because it helps our children and will let us know if they have it or not.

R8: I say it will be a good thing because it will prevent the disease from spreading and affecting our children.

<Files\\FGDs\\FGD-Opinion Leaders- Chaina Assunia-04> - § 10 references coded [12.88% Coverage]

Reference 1 - 1.23% Coverage

R4: as for the testing that will be coming to test for this disease is a good thing because a child can have such a disease that will make him/her cry so much without the parent knowing why. When the testing comes, it will help to detect the disease that will make way for treatment we will gladly receive it.

without delay and we will gladly receive it.

Reference 2 - 0.51% Coverage

R5: we are happy that this testing is coming into our community and we hope that it will help our children to get well in this regard.

Reference 3 - 1.36% Coverage

R10: As for me, I see that it will be a good thing. It will help us not to go all the way to Navrongo for such tastings or visit drug stores so it is a good thing for us.

R3: it will be a good thing that will help us and we will be happy to welcome you. We will be ready to do anything to help you in that regard.

Reference 4 - 1.26% Coverage

R2: we would like to say that Research is doing well because, since time immemorial, they have been working in our communities to help a lot of children which helps them to grow well without falling ill frequently. For this idea to bring a machine into the community, we will say they have done well and God will bless their efforts.

Reference 5 - 1.35% Coverage

R8: as for this, we will even say that we are ready to give you accommodation for you to come to stay with us because you have done extremely well.

You have helped to reduce the cases of malaria among children and if this comes to add up, it means we are developing. You should bring it because that is even what we want, we are very happy with this new idea.

Reference 6 - 1.63% Coverage

R7: as for this machine that you are talking about, we wish it was with us even today. We will be very happy because in the past where there was no testing, you will be sick of one thing and you will be given medication for another which does not cure the sickness. We are very happy with this machine that is coming to us because it will help us know what is wrong with a child and the correct medicine given. Thank you in advance.

Reference 7 - 0.76% Coverage

R4: I want to say that when the machine comes, we will be very happy and whatever assistance is needed from us, we will help whether it is needing batteries or electricity to be charged, we will help.

Reference 8 - 1.56% Coverage

R3: I do not have a question now but I will like to thank you very much because your idea is good as we have all said. What we will ask of you is now that you will truly bring this machine here in reality and we will be praying for that to happen. I also want to say that whatever help you need from us, we will be ready together with our chief and community members to help you with and we will thank you for it.

Reference 9 - 1.82% Coverage

R1: as the chief, I am very happy because you are coming to help my community to have good health and I will not refuse that, so I am happy you are coming into my community to help in that regard. What I ask is that God will give them patience so that they can do their work without well.

In addition, we will talk to the community members not to allow any harm come their way as they come into the community and may God help them in their work. I will like to thank them very much.

Reference 10 - 1.39% Coverage

R7: what I want to add is that we are grateful to you for coming to alert of the help you are bringing into our community so we ask that you fulfill your promise as you have come to share with us. Thank you very much.

R: I know your work is tedious and if you are working because of peoples’ health, God will not let that go in vain, He will help you in your work.

<Files\\FGDs\\FGD-opinion leaders -Mirigu-05> - § 2 references coded [2.28% Coverage]

Reference 1 - 1.60% Coverage

The people selected the Mirigu clinic for the machine because the clinic serves as the head of the six sub-CHPs compounds in the community. Other people were also saying even if the machine is in Navrongo hospital for testing, it is still good. So, now that you are saying it again that the machine will be given to the CHPs compound in the Mirigu community, we are very happy to hear that and I want the day the machine is starting working; they should try and test us first before the children. Because when you start to give the information, you started with the chief palace first and today, you are still sitting here with us; the elders, assemble men, other opinion leaders and religious leaders. So, I think these people with their wives and children should be tested first before any other person.

Reference 2 - 0.68% Coverage

R2: From the information sheet you gave to us, the test is going to be done on children from zero to five years. So, we the opinion leaders should all gather ourselves with our children and wives at the chief’s palace to be tested first before the machine will now be sent to the clinic for the rest of the community members to also test.

<Files\\FGDs\\FGD-Opinion leaders-Chiana Saboro-08> - § 7 references coded [7.69% Coverage]

Reference 1 - 2.03% Coverage

7: When they children from zero to five years are been tested and whose who will be positive, they should test the parents of that child to, this will help. This is the little I have to add.

6: What I have to add is that, you should also include the father because he is the head of the family, it can lead to fight between the mother and the father when she goes to deliver the information to the husband. So, if you can conduct testes to all of as so that it will help us all. These are my last words I will not talk again.

Reference 2 - 0.84% Coverage

I: What If we test only the children.

5: That will not be so helpful. For example, if you want to wet the ground and you spread a rubber before you pure the water how will you achieve your aim so it will not help.

Reference 3 - 0.50% Coverage

9: It is a very good thing it will help us know if our child has the disease. So, when they bring the machine, it will help us.

Reference 4 - 0.52% Coverage

2: Using the machine to do the testing is very good so we pled that they should bring the machine to help those who have the disease.

Reference 5 - 1.05% Coverage

2: We did not even know about the disease and know people have come to tell us how bad the disease is so we will agree for you to use the machine to test to know those who have and to be sure we will get treatment for the disease so that is the reason why we will agree.

Reference 6 - 1.81% Coverage

7: Diong good is not easy, as you people are doing these, we know is a difficult task so, when you return try and tell your authorities to come and help us because if you do not come, we will not have known all these things. VAST has help so many diseases to vanish in the system which existed some time ago. May God blees you for the good work.

We are so happy for your time in one way or the other if we have offended you, please for gave us we are most grateful.

Reference 7 - 0.94% Coverage

3: All I have to say is that whatever you have discuss with as, we all have heard you so, we leave all in the hands God so that he will lead and give as good mind to do the work. We pray that when the work begins the devil will not interact.

<Files\\FGDs\\FGD-Opinion Leaders-Nabango-02> - § 3 references coded [3.37% Coverage]

Reference 1 - 2.58% Coverage

M: How do you think about us doing that?

R10: It will be good to test the children. Because if a child is tested positive, the nurses will advise the parents on how to take care of the child.

maybe they shouldn’t be beating the child or shouting at the child. And you can also report to the child’s teachers about how the child’s condition is so that the teachers will not be involving him in punishments.

M: Excellent, who again?

R6: It will help you know your health status and also will help you to live up to fifty years or seventy years. But if you don’t know about your health status, it can one day attack you and you die. So, we shouldn’t be feeling shy or afraid to go and test. Even those who were having AIDS and didn’t feel shy or afraid to go and test, are still alive and healthy giving birth. So, it will be good for everybody to go and test.

Reference 2 - 0.44% Coverage

R1: It will be good to test the children because if the doctor test and get to know that the child is having that disease, he will help you treat it.

Reference 3 - 0.35% Coverage

R10: It will help people to stop the perception that the research people always come and take our blood to go sell.

<Files\\FGDs\\FGD-with under 5 mothers-Nabango-03> - § 7 references coded [5.57% Coverage]

Reference 1 - 1.08% Coverage

M: What do you think about us doing that?

R6: If they bring that machine here for the test, it will be good for us because you don’t know whether the child or the mother is having the disease.

R9: To me it is good, they should bring the machine for us to know what kind of disease is in our body.

Reference 2 - 0.81% Coverage

R1: To me, it is good they should bring the machine for us to test because some children fall sick frequently and their parents send them to the hospital every day spending a lot of money and increasing their cost of living.

Reference 3 - 0.66% Coverage

R5: If they bring the machine here it will be good because those children that we see grow withered and we are suspecting them to be sickle cell positive can go and test to confirm.

R6: If they bring that machine here for the test, it will be good for us because you don’t know whether the child or the mother is having the disease.

Reference 4 - 0.85% Coverage

R2: It will be good because we will want to know whether your child is having that disease or not

R4: If they are going to test the children, it will be good because we want to know whether our children have it or they don’t have it

Reference 5 - 0.65% Coverage

R5: To me, they should test everybody so that those who are planning to marry will be able to know their sickle cell status and decide whether they can marry or they cannot marry

Reference 6 - 0.72% Coverage

R9: We are just carrying our children but we don’t know whether they have it or they don’t have so if they bring the machine for the testing it will help us to know our children’s sickle cell status

Reference 7 - 0.80% Coverage

R1: To me, it is good because the children are our future leaders so if they bring the machine and test and know that this child is having that disease, they can give medicine to protect the child to live long.

<Files\\IDIs with SCD parents\\IDI-Parent with SCD patient-Doba-01> - § 1 reference coded [1.22% Coverage]

Reference 1 - 1.22% Coverage

R: I think if you people bring the machine to do the testing exercise it will be very good and it will help the people of the Doba community.

M: Why?

R: It will be fine for them to test the children and find out whether they have sickle cell disease or not and those who tested positive will now also attend the hospital for treatment.

<Files\\IDIs with SCD parents\\IDI-Parent with SCD Patient-Korania-07> - § 1 reference coded [3.06% Coverage]

Reference 1 - 3.06% Coverage

I: What do you think about us doing tests to identify children for sickle cell disease with this machine in this community?

R: It will be good.

I: Why do you say it is good?

R: It is good because, if I have my child, I will not know that he/she has a disease but if I happen to go to the hospital and that machine is available for my child to be tested, it will help in taking care of the child.

<Files\\IDIs with SCD parents\\IDI-Parent with SCD patient-Navrongo-02> - § 1 reference coded [0.94% Coverage]

Reference 1 - 0.94% Coverage

R: When you do the test it helps the parents to know their children’s sickle cell status whether they are positive or not.

because you, yourself will begin to know what is wrong with the child.

<Files\\IDIs with SCD parents\\IDI-Parent with SCD Patient-Nawognia-06> - § 1 reference coded [1.05% Coverage]

Reference 1 - 1.05% Coverage

R: It will be very good because the machine will be able to screen the whole body and detect where there is a problem and that will then determine the type of treatment to offer to that person.

<Files\\IDIs with SCD parents\\IDI-Parent with SCD Patient-Paga-05> - § 1 reference coded [1.37% Coverage]

Reference 1 - 1.37% Coverage

R: It is very good.

I: Why do you say it is good?

R: This is good because, if this was closer to me earlier, I would not have suffered the way I did. We move from Dimbasenia to Paga, so if it was close to me my child would not have suffered so much.

<Files\\IDIs with SCD parents\\IDI-Parent with SCD-Pungu-04> - § 1 reference coded [0.90% Coverage]

Reference 1 - 0.90% Coverage

R: considering the kids, I think is a good thing to do so I pray God should help make it come to pass, because the kids are our future. It is a very good initiative.
