# Supplementary material for: Barriers and facilitators to community acceptability of integrating point-of-care testing to screen for sickle cell disease in children in primary healthcare settings in rural Upper East Region of Northern Ghana
Source: PLoS One. 2024 May 20;19(5):e0303520. doi: 10.1371/journal.pone.0303520 (PMC11104616; doi:10.1371/journal.pone.0303520)
Supplement: S1 Data — (ZIP) [file pone.0303520.s001.zip › S1_Data for community members/D Views on CHOs doing the screening.docx]

**Name:** Agree for CHOs to do the screening

<Files\\FGDs\\FGD with under 5 mothers-Chiana Yidania-07> - § 2 references coded [2.23% Coverage]

Reference 1 - 1.31% Coverage

R1: as for me, I would agree or let me say I am ok with it because when my child is sick and they ask me to go to Navrongo for treatment, it would come as stress to me so if it is going to be with them and here with us, I will agree. Thank you

R2: I would also say yes and also add that if some of you would join in the testing, it would also be good

Reference 2 - 0.92% Coverage

R5: if it’s the nurses here who are going to do it, that will be nice because we have been with them here and have also come to them for our normal ANC and we are familiar with them. So, I think it would be good if they are those doing the testing.

<Files\\FGDs\\FGD with under 5 mothers-Chiana-01> - § 1 reference coded [3.85% Coverage]

Reference 1 - 3.85% Coverage

I: Who has any response to add? So, would you agree that this machine be used to test your children when you come for the normal weighing? If yes, why and if no why?

R2: we would agree because your child can wake up and be sick and you would not know what is wrong with the child, so when they test it will be good.

R8: I will agree because you cannot be walking with a sickness and then you get the treatment and you say you do not want again, why? So as for me I will agree to test my child so that if she is sick of the disease, she can get treatment because we do not hide a disease. Thank you.

I: Who has an addition?

R9: why will you not agree when someone has come to help me with something I cannot do myself, why would I say no? why will I not agree because I am in the world and also need help from others. The children we birth will also grow to develop the society so your child with that sickness gets treatment and become well, is it not better than if she dies? I know it is good news and we will have to accept it with open arms. This is my take.

<Files\\FGDs\\FGD with under 5 mothers-Mirirgu-06> - § 7 references coded [5.24% Coverage]

Reference 1 - 0.74% Coverage

M: Will you agree for the CHOs to use the device at the CHPs compound/clinic level to test your children for sickle cell disease?

Rs: Yes

M: Why will you agree?

R4: Because it is going to help our lives and all the people in the Mirigu community.

Reference 2 - 0.70% Coverage

R9: It will bring help to all Mirigu people because, we will come and test and if we have it, the doctors will help us treat it well.

R2: If they bring the machine for the testing, I think it will help reduce the spread of the disease.

Reference 3 - 0.74% Coverage

R1: It is good they should use the nurses here in the CHPS to be doing the testing because we are already here with them. So, if there is any problem, they can easily talk to us about how we will go about it and we can also easily understand them too.

Reference 4 - 0.92% Coverage

M: Do you have trust in the nurses here that they can be able to do the work?

R2: Yes, we trust them because we have been doing our things with the most time, so we have that trust that they can use the machine and test us and will not spread our sickle cell information to everyone.

They have confidentiality.

Reference 5 - 0.85% Coverage

R4: I think the nurses are the appropriate people to do the test because they have been trained and have the skills of doing it.

R3: The nurses are the appropriate people if they learn more about how to use the machine in addition to the skills that they have and it will be very good.

Reference 6 - 0.72% Coverage

R10: I think it is very good to use the CHPs nurses to do the test because they are there with us and they have been taken care of our health need at the community.

when we are pregnant till we give birth. So, they know most of us our lifestyles and the way they will handle us when your child tested positive.

Reference 7 - 0.57% Coverage

R6: I think the CHPs nurse deal with us from day one up to date so if they are doing the test, it will be the best and children’s mothers will be willing to come in their numbers for testing.

<Files\\FGDs\\FGD-Opinion Leaders- Chaina Assunia-04> - § 3 references coded [4.12% Coverage]

Reference 1 - 1.23% Coverage

R4: as for the testing that will be coming to test for this disease is a good thing because a child can have such a disease that will make him/her cry so much without the parent knowing why. When the testing comes, it will help to detect the disease that will make way for treatment without delay and we will gladly receive it.

Reference 2 - 1.86% Coverage

R8: we will agree because we will not even know how to use the machine. If it comes to our community, we will be required to wash our hands with soap before using and where will be get that from. Our only issue with be the fact that some mothers complain of neglect by the nurses who concentrate on their phones. Help us to beg them as we have already been doing because they are educated about health in order to help us. We will like them to use the machine and use it well to take care of us.

Reference 3 - 1.02% Coverage

R7: we agree that the nurses working here should use the machine because there is a saying in Kasem that ‘‘those who do not know the work cannot do it’’. It is the nurses who will know how to use the machine and that is why we are agreeing for them to use it to help us.

<Files\\FGDs\\FGD-opinion leaders -Mirigu-05> - § 3 references coded [3.31% Coverage]

Reference 1 - 1.67% Coverage

R6: Yes, we will agree for them to use the machine and test our children. Also, we have people that are educated in this community. So, I suggest if you can select some of these people to also be helping the CHOs in the testing exercise it will be good because using the nurses alone to do the work, I think it will increase their workload. Maybe you can select some of us the community volunteers to help in the testing.

R5: To me, I think this is a highly technical professional and it concerns people’s health. So, if you make a mistake and take ignorant human beings to go and do this work, they will end up killing people and ignoring telling people that they have got it when they haven’t got it. So, for me, I will say that they should use the nurses that are already in the profession to do the work and it will be good for us all.

Reference 2 - 1.41% Coverage

R8: To me, we will agree for the CHOs to use the machine and test the children. And what I also want to say is that; if something like this is coming to the community clinic, all the people will accept it but when it gets to the time it will start to work, no one is there to work. So, the research people should tell the nurses to be serious in this work so that our people too will be ready to bring their children for the test. We don’t want to accept and the machine will come and if we go to test, we will be receiving complaints from the nurses that itis because this happened and that is why we don’t test again, we don’t want it to happen that way. We want a continuous and smooth running of this work.

Reference 3 - 0.23% Coverage

R4: From zero to five years, I think it is good but if they can extend it to age six, I think it will be very good.

<Files\\FGDs\\FGD-Opinion leaders-Chiana Saboro-08> - § 1 reference coded [1.71% Coverage]

Reference 1 - 1.71% Coverage

2: You will not be able to know some once mind because they are at their work place as to whether they will spread the news or not I will not be able to know that, because the kind of mind set you as a person have regarding the questions you are asking me now is something I did not know you will be asking me so when the testing starts and they get to know I have the disease I will not be able to know if they will spread the news or not.

<Files\\FGDs\\FGD-Opinion Leaders-Nabango-02> - § 3 references coded [3.28% Coverage]

Reference 1 - 2.08% Coverage

M: Will you agree for the CHOs to use the device to test your child for sickle cell disease?

R5: Yes, we will agree because it is going to help us to know whether we have that disease or not

R8: We will agree but before that, we have to let our women that are sending the children to the hospitals know the importance of that test. Because some women if they are going to take their children’s blood samples for a test, they don’t always want to agree. Saying that my child’s blood is not enough and they want to take the blood again for a test, I will not agree. So, whenever the women bring their children to the hospitals, the nurses should try to educate them on the importance of the test.

Reference 2 - 0.30% Coverage

R10: It will be good but before that, there should be proper counseling for the parent on the disease.

Reference 3 - 0.89% Coverage

R2: The women are already used to the nurses that are here. If you are going to bring people from a different place, it may be difficult for the women to become used to them.

So, if the nurses here don’t know how to use the machine, then they provide them with training before the whole work starts.

<Files\\FGDs\\FGD-with under 5 mothers-Nabango-03> - § 5 references coded [6.00% Coverage]

Reference 1 - 2.05% Coverage

M: Will you agree for us to use the nurses in the health centers in your community to use the machine and test the children?

R5: If you let the nurses here test it will be good because if they are coming to our communities to weigh our children, they can just add the testing which is better than going to the hospitals for the test. The hospitals normally delay before they will give you your results. Sometimes it will delay up to five years before they will start looking for the person with the results and by the time they get him, he is already a dead person. (FGD-with under 5 mothers-Nabango-03)

Reference 2 - 1.48% Coverage

R9: We are d to the nurses that are here so if you give them the machine to test our children, I think there will be no misunderstanding between the nurses and us but if it is going to be different people that will use the machine and test then there will be difficulties in understanding one another

R7: If you give it to the nurses that are here it will be better than other nurses from a different place

Reference 3 - 0.54% Coverage

R4: We do most of our health activities with the nurses that are here so if you let them use the machine and do the testing it will be easy for us

Reference 4 - 0.86% Coverage

M: Do you trust the nurses in your health centers that are to use the machine for the testing?

R6: For the work to do well, you should give the machine to the nurses that are doing the weighing they politely talk to us but not any nurse

Reference 5 - 1.06% Coverage

R10: We want those nurses here that have been taking care of our children to use the machine and test our children for us

R4: Give the machine to our nurses that weigh our children here but don’t take nurses from anywhere to do the testing, I think this will help the testing to be done well.

<Files\\IDIs with SCD parents\\IDI-Parent with SCD patient-Doba-01> - § 2 references coded [4.10% Coverage]

Reference 1 - 2.23% Coverage

R: They will agree for the CHOs to do the testing because looking at it, sickle cell disease is also a killer disease that kills a lot of people, especially children so if they bring the machine to test for sickle cell disease then it will be very good.

M: Why?

R: They will agree because when you have disease in your system and it affects you a lot. You will not sit down and say that you will not go to the hospital for medical treatment and even if you go to the hospital. They will test you again to confirm whether it is true that you have the disease or not before, they will put you on medical treatment

Reference 2 - 1.87% Coverage

M: Good, what concerns do you have with using CHPs nurses to test for sickle cell in children?

R: My concern is that if you people bring the device for testing the CHPs nurses should also do the testing well for the benefit of all.

M: Okay, what concerns about trust in the CHPs nurses to appropriately test for sickle cell?

R: I think some of the nurses are not good that is what I cannot say much but those who are well-trained nurses with better qualifications can do the testing to come out with good results.

<Files\\IDIs with SCD parents\\IDI-Parent with SCD Patient-Korania-07> - § 1 reference coded [2.64% Coverage]

Reference 1 - 2.64% Coverage

I: Will you agree for the CHOs to use this machine to test your child for sickle cell disease?

R: Yes

I: Why?

R: As I said earlier on, if I do not send my child to the hospital, I will not know what is wrong with the child. But if I send my child and the nurses use the machine to test, they will be able to know what is wrong with my child.

<Files\\IDIs with SCD parents\\IDI-Parent with SCD patient-Navrongo-02> - § 1 reference coded [0.49% Coverage]

Reference 1 - 0.49% Coverage

M: Will you agree for the CHOs to use the device to test your child for sickle cell disease?

R: Yes

<Files\\IDIs with SCD parents\\IDI-Parent with SCD Patient-Nawognia-06> - § 1 reference coded [1.71% Coverage]

Reference 1 - 1.71% Coverage

I: Will you agree for the nurses to use the machine to test the children?

R: Why not, I will agree.

I: Why will you agree?

R: Because I know it will be of help to the children. If it will bring distractions, they will not have brought it, the machine is of good help that is why they brought it to test children.

<Files\\IDIs with SCD parents\\IDI-Parent with SCD Patient-Paga-05> - § 1 reference coded [1.40% Coverage]

Reference 1 - 1.40% Coverage

I: Will you agree the nurses at the clinics to use this machine to do the testing?

R: There is no problem about that even if the nurses I stay with can do it, I do not have a problem.

I will even like it more and the nurses are even two, a man and a woman.

<Files\\IDIs with SCD parents\\IDI-Parent with SCD-Pungu-04> - § 1 reference coded [1.11% Coverage]

Reference 1 - 1.11% Coverage

R: Yes, it will be good if they will be able to add it to their work, especially we those who are close will be able to reach there when there is an attack in the night. So, it is good for them to use it.
