# Supplementary material for: Barriers and facilitators to community acceptability of integrating point-of-care testing to screen for sickle cell disease in children in primary healthcare settings in rural Upper East Region of Northern Ghana
Source: PLoS One. 2024 May 20;19(5):e0303520. doi: 10.1371/journal.pone.0303520 (PMC11104616; doi:10.1371/journal.pone.0303520)
Supplement: S1 Data — (ZIP) [file pone.0303520.s001.zip › S1_Data for community members/E Factors that could affect acceptability of the screening.docx]

**Name:** Factors affecting acceptability of the screening exercise

<Files\\FGDs\\FGD with under 5 mothers-Chiana-01> - § 5 references coded [7.52% Coverage]

Reference 1 - 1.61% Coverage

R8: what I know is that when it comes and then they start discriminating among us. When others come they treat them well but charge others. You can imagine what those who are charged will say when they return home to their families. This can let people to lose hope in bringing their children for the testing and maybe those children will have the disease. When they charge 5 cedis and later on it goes to 10 cedis, this will discourage people.

Reference 2 - 2.64% Coverage

R9: when it comes to things like this, we all have to agree to accept some of this assistance. Some people will not even come whether money is required or not, to bring their child for the testing. Secondly, the economy is harsh and we do not have money. When they start charging the 2 cedis, we can manage and get it but when the charge keeps increasing, they will now be dodging and cannot pay for the testing even though the child might have had the sickness. Sometimes when you do not have the 10 cedis to pay, the child’s sickness may be increasing which can lead to a severe attack that can cause death. So, when it comes, we beg that you let it be affordable so that we can get our children tested in order to get treatment.

Reference 3 - 1.24% Coverage

R6: I want to know if you will bring people from outside to come and test or if the nurses that are at the various communities will be testing the children. This is because most of them despise us because we do not look rich when we come there, so when those who dress and look rich come then they treat them or attend to them first before us.

Reference 4 - 1.28% Coverage

I: So, is there anything religiously that you think will prevent this program from happening?

R3: to me I think there is nothing like that in my religion that will not allow us to take part in the exercise because is not ‘juju’ that they are bringing to help everyone and everybody whether Christian or Muslim, we all fall sick and we need treatment so I don’t think there is going to be something like that.

Reference 5 - 0.76% Coverage

R9: culturally, it cannot prevent it because if you are not alive you cannot perform tradition and you are coming to give us health assistance but not bad influence, so I do not think that can prevent this.

<Files\\FGDs\\FGD with under 5 mothers-Mirirgu-06> - § 7 references coded [5.51% Coverage]

Reference 1 - 0.68% Coverage

R6: Some people too will also go to where they are doing the test with a lack of understanding which can lead to a fight. And if there is a fight at the testing point, the vulnerable people will not like to go there again and test.

Reference 2 - 0.75% Coverage

M: What are the factors in this community that are likely to affect the acceptance of the sickle cell disease testing exercise in this community?

R8: Fears of being tested positive can make people not to go and test. Most people will be having fears that; if they test and they are positive what are going to do.

Reference 3 - 0.73% Coverage

R1: With the traditional believers, some of them don’t like going to the hospital, and the Christians too, there is this deeper life church, they don’t go to the hospital when they are sick. So, the testing like this, they will not agree to come.

Reference 4 - 0.90% Coverage

R9: It can also bring misunderstanding between you and your husband. When the husband learns that the child is having that disease, he will now be thinking that it is the mother that transmitted it to the child. This will happen if the husband doesn’t have an understanding of the disease from the nurses.

Reference 5 - 0.29% Coverage

R3: Others will also firmly believe in their gods to heal them than to go to the hospital and test.

Reference 6 - 1.10% Coverage

M: Good, what are the cultural beliefs and factors that are likely to affect acceptance of the sickle cell disease testing exercise in this community?

R9: I don’t think there are cultural beliefs in this community that affect the acceptance of the testing. Although, we have some families that don’t send their children to hospital apart from the traditional healers alone.

Reference 7 - 1.04% Coverage

R5: For me alone, I don’t think there are cultural factors that can prevent the testing of the sickle cell because nowadays everybody sends his/her children to the hospital for treatment. Unless they try and it didn’t work then they will say that its spiritual sickness. So, let us go to the traditional healers or our local herbs for treatment and see.

<Files\\FGDs\\FGD-Opinion Leaders- Chaina Assunia-04> - § 4 references coded [4.53% Coverage]

Reference 1 - 1.78% Coverage

R7: what we will have to do is to trust that the people you (Ghana VAST) will choose to do the testing will not leak the information. I think if I am tested and whatever the results may be, if it does not go out, it cannot spread like the wind to other people. If the results are leaked, someone who has the same disease might keep quiet about the whole situation which can kill the person and that is not go enough. This is what will help for the testing to move on well.

Reference 2 - 1.31% Coverage

R8: what will impede the implementation of this excise will be if you (VAST) do not come to work in truth. To get medications to cure a disease is not a barrier to us, we do not have traditions or churches that do not allow blood transfusion here. If only that your workers do not work in truth, we will surely sack you. This is what I have seen.

Reference 3 - 1.06% Coverage

R7: what I can see as an impediment will be if you are here and not working with patience but being lazy.

R7: If you come clear in your work, the various community sections will support your in this exercise, but there are not traditional practices or church that can impede this project.

Reference 4 - 0.38% Coverage

R4: what can impede the work is when you machines get spoiled and you cannot test the children again.

<Files\\FGDs\\FGD-opinion leaders -Mirigu-05> - § 4 references coded [3.02% Coverage]

Reference 1 - 1.15% Coverage

R2: If there is poor information and communication with the community people. If the community people do not get the right information about how the whole work is going to be and how helpful it is, I think they will not be willing to come and test*.*

So, it is going to be a major factor that can affect the acceptance of the exercise.

R6: If there is stigmatization in the community. When a person is tested positive and the community people are stigmatizing the person, I think other people that have not tested will not agree and test because they will also be stigmatized.

Reference 2 - 0.61% Coverage

R4: If it also involves payment of money, so many people will not agree to go and test because most of us here are very poor. People will be saying research people have brought a machine to take the little money that we have away. And they will not accept and use all their last wealth and come and test.

Reference 3 - 0.82% Coverage

M: For religious beliefs?

R9: The religious believers that may not accept this testing will be the Jehovah’s witnesses and these Jehovah’s witness people are not in this community. Last year those who were refusing to take the CSM vaccine, the chief forced them and they all vaccinated. So, apart from the Jehovah’s witness, there is no other Christian or traditional body that will not accept and do this test.

Reference 4 - 0.45% Coverage

R3: There is no single person in this community that will say he or she will not go and test simply because his or her church or tradition doesn’t allow them to go to hospitals. In this community, we are all serving one God.

<Files\\FGDs\\FGD-Opinion leaders-Chiana Saboro-08> - § 2 references coded [1.56% Coverage]

Reference 1 - 0.72% Coverage

1: If you come to cure us, it is very good but your machine will be the problem if it is movable to move it from the big hospital to the small once because we cannot all use one machine.

Reference 2 - 0.84% Coverage

I: As leaders of the community, what do you think can stop this initiative from becoming a success, regarding church and traditional believes?

9: This is help you have offer so nothing can stop it from happening.

<Files\\FGDs\\FGD-Opinion Leaders-Nabango-02> - § 7 references coded [7.79% Coverage]

Reference 1 - 0.93% Coverage

R1: To me, if the test is going to involve payment of money, it will be a challenge for parents to send their children for testing.

R5: If the machine that is going to use and do this testing exercise is going to be taking a long time before giving out the results, I think it is going to be another challenge.

Reference 2 - 0.94% Coverage

M: What religious beliefs could affect acceptance and testing for sickle cell in your community?

R4: The religious beliefs that don’t allow doctors to take a patient’s blood for testing or don’t allow a sick person to go to the hospital exist but, not in this our community here. Not even at our traditional level.

Reference 3 - 1.50% Coverage

R8: This religious belief is there in this community. A week ago, a man brought his wife here to the hospital; the woman’s BP was very high. They referred the woman to Navrongo hospital, but the man said his wife is not sick she will not go anywhere and was seriously praying for the wife on the sickbed. So, I think the religious beliefs that could affect this test are there here in our community. For the church aspect, it is there but, to our traditional beliefs, we don’t have such a thing here.

Reference 4 - 0.75% Coverage

R9: To me, I think the religious beliefs will not be a challenge like the illiteracy rate here. During the counseling and if they don’t interpret it in the local language well for the person to understand, I think it will bring some little challenges.

Reference 5 - 1.41% Coverage

R10: Nowadays the traditional beliefs are not more like the olden days when they only want to use only local treatment for the person. Now, they accept and use both the local and the hospital medicine for treatment. But it is now the church that believes that; it is God who created life and He is the only one who heals sickness. And others too, if they don’t want to come out for the test maybe it is because they don’t want to leave their business and go for the test.

Reference 6 - 0.69% Coverage

R3: As for the religious beliefs that could affect the testing they are there. We have a church that does evangelism; telling people not to receive blood from hospitals whenever they are sick. That it is only God that gives blood.

Reference 7 - 1.57% Coverage

R3: The traditionalist has now agreed and added hospital treatment to their treatment. But in the olden days, a woman can be in labor to give birth but they will say she will not go to the hospital, they have to consult the gods before she will go to the hospital. But now, the woman will go to the hospital and they too will be going to consult their gods, no more waiting to consult the gods before the woman can now go to the hospital. So, the traditionalist also accepts hospital treatment in addition to their treatment.

<Files\\FGDs\\FGD-with under 5 mothers-Nabango-03> - § 4 references coded [4.12% Coverage]

Reference 1 - 1.60% Coverage

R9: For some people, it is a taboo to take his or her blood and if such a person refused and removes his blood, they normally say he will not be part of the tradition again

R2: Some church believes that their member’s blood should not be taken even when they are to do a test and if a member refuses and take his blood out for a test, they will stop that person from going to that church. Example SDA church and Jehovah people. is another example

Reference 2 - 0.64% Coverage

R1: Some of our traditional homes, normally say that the child needs a ring but not a hospital treatment so they don’t always want to bring the child to the hospital for a test

Reference 3 - 1.49% Coverage

R5: Some of our homes too, don’t allow their child to go to hospital anytime the children are sick. For example, one child, since they gave birth to the child, they never brought the child to the hospital so one day the child was seriously sick and they brought the child to the hospital and the doctors injected every part of the child with water and brought the child to life. If not, the child would have died

Reference 4 - 0.38% Coverage

R: Some will also say that the child is a witch and you are still sending her to the hospital to do what?

<Files\\IDIs with SCD parents\\IDI-Parent with SCD patient-Doba-01> - § 1 reference coded [3.06% Coverage]

Reference 1 - 3.06% Coverage

R: I think some people are there when it happens to their child, they will not go to the hospital but he/she will be roaming looking for traditional rituals and herbs for treatment. They are doing this with the perception that their ancestors and grandparents will heal their child’s sickness.

Some religious beliefs also believe that God is the healer of everything so they will not go to the hospital but rather keep on praying for their God to heal their child who is a sickle cell patient. All those people don’t believe in in-hospital treatment at all apart from their religious beliefs. Some people are also willing to go to the hospital for treatment so those are the factors that will affect the acceptance of the sickle cell disease testing exercise in this community

<Files\\IDIs with SCD parents\\IDI-Parent with SCD Patient-Korania-07> - § 1 reference coded [1.82% Coverage]

Reference 1 - 1.82% Coverage

I: What factors do you think can affect the acceptance of this exercise in this community?

R: there is nothing like that.

I: What religious or cultural factors can affect the exercise?

R: there is nothing that can affect this program.

<Files\\IDIs with SCD parents\\IDI-Parent with SCD patient-Navrongo-02> - § 2 references coded [2.69% Coverage]

Reference 1 - 0.81% Coverage

M: What factors do you think are likely to affect the acceptance of the sickle cell disease testing exercise in this community?

R: Please for that one, I don’t know.

Reference 2 - 1.88% Coverage

M: Okay, do you think there are religious beliefs or cultural beliefs that are likely to affect the acceptance of the sickle cell disease testing exercise in this community?

R: I think some people will think that when they test and it is positive, what will they do? So, in this case, they decide not to test so that they will be living their normal life without any fears.

<Files\\IDIs with SCD parents\\IDI-Parent with SCD Patient-Paga-05> - § 2 references coded [2.70% Coverage]

Reference 1 - 0.81% Coverage

I: What factors do you think are likely to affect the implementation of this exercise?

R: I do not think there is any factor, none that I know of.

Reference 2 - 1.89% Coverage

I: What religious or cultural factors can affect this project from being implemented successfully?

R: I do not think there is something like that, because this is not something that prevents anything traditionally from happing or goes contrary to any Christian belief but rather giving life. So, I do not think this would have any effect on them.

<Files\\IDIs with SCD parents\\IDI-Parent with SCD-Pungu-04> - § 1 reference coded [1.02% Coverage]

Reference 1 - 1.02% Coverage

I: Okay. What do you think can prevent the implementation of this project from becoming a success?

R: I do not think there is something that can prevent this project to become a success.
