# Supplementary material for: Barriers and facilitators to community acceptability of integrating point-of-care testing to screen for sickle cell disease in children in primary healthcare settings in rural Upper East Region of Northern Ghana
Source: PLoS One. 2024 May 20;19(5):e0303520. doi: 10.1371/journal.pone.0303520 (PMC11104616; doi:10.1371/journal.pone.0303520)
Supplement: S1 Data — (ZIP) [file pone.0303520.s001.zip › S1_Data for community members/F Suggestions to improve acceptability.docx]

**Name:** Suggestions to improve acceptability

<Files\\FGDs\\FGD with under 5 mothers-Chiana Yidania-07> - § 3 references coded [2.91% Coverage]

Reference 1 - 1.42% Coverage

R4: One thing I want to talk about is time, we should make it in the morning because some of us have husbands who do not understanding things and if we come here in the evening and we are not able to cook at home, then we might even be stopped from coming for the exercise. (FGD with under 5 mothers-Chiana Yidania-07)

to this place.

So I think we should get a specific day in the week for this so that it will make things easy and good for us. Thank you

Reference 2 - 0.78% Coverage

R8: what I also have to say is that we are getting to the farming season, so if you could make it in the morning so that we can come and go back early, that will help us and we can do other farming activities. (FGD with under 5 mothers-Chiana Yidania-07)

Reference 3 - 0.71% Coverage

R5: I think we should look through the week and select one day and use that day for the activity, so that it won’t interrupt our work especially now that we are getting to the farming season.

<Files\\FGDs\\FGD with under 5 mothers-Chiana-01> - § 5 references coded [6.59% Coverage]

Reference 1 - 1.85% Coverage

R7: I think what should be added is we the mothers should avail ourselves when we are called upon, we should try as much as possible to show up so that they will know we are serious. (FGD with under 5 mothers-Chiana-01)

and not get discouraged. Because if we do not show up, next time we will not be given a chance.

R3: what I think is that, for instance my child test positive and I do not have money to go the hospital when referred, you can help us with a little amount to enable us take the ward to the hospital which will be good help. Thank you.

Reference 2 - 1.50% Coverage

: So what help do you people have add to the successful implementation of this program? I earlier on asked this question but I want to ask it again?

R3: I think we the mothers should come out when you call us because when you call the first day and we come out in our numbers, you will be happy.

But if we do not come out and for the second time too we do not come, then you will be forced to redraw your services.

Reference 3 - 1.06% Coverage

R8: I also think what can help is we the mothers should report on time when called upon so that it will encourage you to help us more. If you ask us to come at 8am but we end up coming at 11am, it will discourage you and that will show that we are not serious with thw matter which is urgent.

Reference 4 - 1.41% Coverage

R9: I think when we get home and share this discussion with our families and they refuse it, that is where it will not go well. But if the family agrees, then they need to support us in all we do to make it a success. So if you are asked to gather in the future, for such an educative activity where children will be tested to know their disease status, they will understand and support you.

Reference 5 - 0.77% Coverage

R7: I think the family needs to be concerned because when the child is sick it affects the whole family so they need to help us so that when the child is treated there will be time for other thinks in the family.

<Files\\FGDs\\FGD with under 5 mothers-Mirirgu-06> - § 4 references coded [7.23% Coverage]

Reference 1 - 1.24% Coverage

M: What are your solutions to solve these factors that are likely to affect the study?

R9: To me, they should look for medicine for it so that if you come and test and confirm that you are positive then they can prescribe that medicine for you, and you will take and it will treat that disease and you will know that you don’t have it again. So, if it is in this way, I think the problem will solve in the community.

Reference 2 - 2.75% Coverage

R2: We should pre-inform the community people before the whole exercise. The community people should be educated on the disease and let them know that if you have that disease, it doesn’t mean you are going to die. (FGD with under 5 mothers-Mirirgu-06)

Inform them that there is medicine for them to be taking that will let them stay healthy in their lives. But if you don’t tell them this and you rather tell them if they don’t come for the test and you are having that disease, you are going to die, it is not going to bring changes. But if you talk to them that if they come and test and they are positive, you will give them medicine to take and the disease will go, and even if you test negative, they can vaccinate you against it. If you talk to them like this, I think they will not be having fears in them and they will agree and come out for the test. But if you don’t give them good education on the disease, the people will not come out and do the testing.

Reference 3 - 2.44% Coverage

M: What are your suggestions to make this study work in your community?

R3: To me, the nurses that are in our community here should go out and talk to the people to come and have a meeting with them. I think with this, the community people will be able to understand them and come out for the screening. (FGD with under 5 mothers-Mirirgu-06)

and they will have that discussion with them.

R9: We those who are for this discussion, are the representatives of the whole Mirigu people in this discussion. So, if we go back to our various homes, we should try and talk to other people about what we have discussed here so that they too will get to know what the research people are planning to do for the community. With this, if you are going to start the testing exercise and you call them to come, they will be interested to come for the test. (FGD with under 5 mothers-Mirirgu-06)

or any information you have for them.

Reference 4 - 0.80% Coverage

R1: We also have to be going to the information center to announce and educate the people before the whole work starts. (FGD with under 5 mothers-Mirirgu-06)

R2: I think if the assemble man and the chief talk to the community people, I think the community people will respect them and come out for the test. (FGD with under 5 mothers-Mirirgu-06)

<Files\\FGDs\\FGD-Opinion Leaders- Chaina Assunia-04> - § 4 references coded [5.54% Coverage]

Reference 1 - 1.78% Coverage

R7: what we will have to do is to trust that the people you (Ghana VAST) will choose to do the testing will not leak the information. I think if I am tested and what ever the results may be, if it does not go out, it cannot spread like the wind to other people. If the results are leaked, someone who has the same disease might keep quiet about the whole situation which can kill the person and that is not go enough. This is what will help for the testing to move on well.

Reference 2 - 1.67% Coverage

R10: I do not think it will be a problem to we seated here but maybe those left out, but as for those of we mothers here, we are even happy with the project.

R7: we know that VAST has always done their work with patience, so if they (VAST) work with more patience, it will help and everyone will accept the project. (FGD-Opinion Leaders- Chaina Assunia-04)

In addition to they using all the resources they have to do the work, that will go a long way for the work to implemented well.

Reference 3 - 1.13% Coverage

R8: what we can do is to always remind and encourage the mothers to bring the children to the clinics and hope that they follow you rules and regulations, so that you can test the children for us to know whether they are sick or well. We are ready to let all the children come out in their numbers.

Reference 4 - 0.96% Coverage

R4: I think we can advise that you should always pre-inform us early so that we can also remind the mothers about it a day or two before your visit. This is because we do not sit down every day, so if we are aware ahead of time, we can organize ourselves.

<Files\\FGDs\\FGD-opinion leaders -Mirigu-05> - § 3 references coded [2.58% Coverage]

Reference 1 - 0.63% Coverage

R2: What I will like to add is that; the research people should also have a strong campaign to influence people to go and test and know their sickle cell status. (FGD-opinion leaders -Mirigu-05)

which will also help partners to know whether they can marry or they cannot marry. I think this will help prevent people from getting it or spreading it.

Reference 2 - 0.68% Coverage

R2: Most of our people here are ignorant about this disease. So, if you could let us have a general public gathering so that you educate them more on the disease and what you are planning to do for them concerning the sickle cell disease. So, I think if you do it this way, it will help more people to understand the importance of the test and the need for them to take part. (FGD-opinion leaders -Mirigu-05)

Reference 3 - 1.27% Coverage

R9: I want to also say the same thing, but I think there should be a durbar that everybody should be involved. The children, the opinion leaders, mothers, and even community associations should be present in the durbar so that it will be like it is a festival celebration. Drumming and dancing and all the chiefs and the sub-chiefs will be present, it will involve all the twelve sub-communities in Mirigu. And at the end, then the research people will now come and deliver the information to the gathering. We will just launch it and kick off the study. I think with this, the information will be able to reach everybody in the community.

<Files\\FGDs\\FGD-Opinion leaders-Chiana Saboro-08> - § 2 references coded [1.26% Coverage]

Reference 1 - 0.58% Coverage

I: What can we do to convince parents and family to accept this study work?

2: I think we will have to spread the massage and educate them very well

Reference 2 - 0.68% Coverage

7: Am sure if we pass through the churches to deliver this massage it will spread faster. (FGD-Opinion leaders-Chiana Saboro-08)

and me as a pastor,

I will say it in my church and encourage other pastors to do same.

<Files\\FGDs\\FGD-Opinion Leaders-Nabango-02> - § 1 reference coded [1.68% Coverage]

Reference 1 - 1.68% Coverage

R4: I will like to say, they should try and get community volunteers and train them so that they serve as people that will be talking to the community people on sickle cell disease and also responsible for organizing sickle cell children for vaccination and health talks. (FGD-Opinion Leaders-Nabango-02)

R8: Even those volunteers that my brother is talking of, they can be the best people to go around the community talk to people about the exercise and that will improve patronage

and get you other children that are having that disease but not necessarily only those children that are from zero to five years.

<Files\\FGDs\\FGD-with under 5 mothers-Nabango-03> - § 10 references coded [10.37% Coverage]

Reference 1 - 1.22% Coverage

M: What are your suggestions to make this study work in your community?

R5: To me, I suggest that they should give the machine to the nurses in our community for the homes that don’t send their children to the hospital so that they can also come for the test. Because they don’t go to hospitals, they can come to the community and test

Reference 2 - 0.48% Coverage

R7: To me, I say we should educate those who don’t go to hospitals on this disease what it is and why they need to come for the test

Reference 3 - 1.66% Coverage

R10: There was a day that some people came here for vaccination excises but because they were not our community nurses, people refused to come out for the vaccine. But whenever our community nurses call us for vaccines or anything, the community people normally come out in their numbers. (FGD-with under 5 mothers-Nabango-03)

So, I think our community people trust and like our community nurses’ work more than any other outside persons so the machine should be given to our community nurses here

Reference 4 - 2.04% Coverage

M: What suggestions do you have to make for families to accept and bring their children for the testing?

R2: They should add the testing exercises to the weighing days because whenever we have weighing exercises, the nurses normally inform us that this day we should come for weighing. So, I think after the weighing then they will just test your child. (FGD-with under 5 mothers-Nabango-03)

R9: To me, I suggest that they should always tell us that there is a general meeting but if they say it is a testing exercise, the community people will say it is a useless thing so they will not come out.

Reference 5 - 0.91% Coverage

R3: To me, they should say that; it is a visitor that comes to meet women with children. I think everybody will come out because they don’t know what the visitor is having for them so they will want to come out and know what the visitor has for them.

Reference 6 - 1.41% Coverage

R6: We have something here in our community that is called the mother-to-mother group from world vision so if they said the mother-to-mother group is having a meeting, I think everybody will come out and we just have it done. This is because the world vision normally share food with this mother-to-mother group that is why if you say mother to mother meeting, they all want to come out.

Reference 7 - 1.27% Coverage

M: What are your suggestions relating to disclosing results?

R4: If your child is diagnosed to be sickle cell positive, the nurse should call you on phone to come and she can then advise you before telling you your child is having that disease. I think with this nobody will get to know that the nurse called you and said something like that to you

Reference 8 - 0.27% Coverage

R9: Calling you alone and informing you is better than telling you publicly

Reference 9 - 0.45% Coverage

R6: Also, to those who are doing the testing, they shouldn’t be treating those whose children are having this disease badly

Reference 10 - 0.67% Coverage

R5: What I have to add is the nurses, should always try and exercise patience and politely explain to us so that even if your child is having the disease, there will not be fear in it.

<Files\\IDIs with SCD parents\\IDI-Parent with SCD patient-Doba-01> - § 4 references coded [7.19% Coverage]

Reference 1 - 1.76% Coverage

M: Good, what other suggestions would you make to improve this study or the sickle cell testing exercise in your community?

R: I think everything is that we should pray hard so that if God willing and you people are coming to do the testing exercise everybody will agree and come out in numbers with their children for testing of sickle cell disease. All of the community members will be in one point of view to accept the exercise and forget about soothsayers and religious beliefs.

Reference 2 - 2.07% Coverage

M: What suggestions do you have about acceptance by the mothers and families to improve this study or the sickle cell testing exercise in your community?

R: What I have to say is using my experience in testing to find out my child’s sickle cell status has helped me a lot because after testing I now know that my child is a sickle cell patient. For now, I know where to carry my child to when he falls sick so if parents or families can test and know their children’s status, it will help them and improve their families/parents’ health conditions with their children.

Reference 3 - 1.40% Coverage

M: Excellent, what suggestions do you have about the use of CHPs nurses to test for improving this study or the sickle cell testing exercise in your community?

R: When the nurses are doing the testing, they should continue telling the people the importance of the exercise to them and their children so the community will come in numbers for the testing exercise to be done perfectly. (IDI-Parent with SCD patient-Doba-01)

Reference 4 - 1.96% Coverage

M: Okay, what suggestions do you have about disclosing results for improving this study or the sickle cell testing exercise in your community?

R: I think when the results come and it is positive, they should say it in a way that will encourage the person to know how to handle the sickness with the medical advice so that the person will not be thinking about the death of the child. Telling them what to do when it attacks the child and by sending him/her to hospital for medical treatment and how it improves the child’s health condition.

<Files\\IDIs with SCD parents\\IDI-Parent with SCD Patient-Korania-07> - § 1 reference coded [2.09% Coverage]

Reference 1 - 2.09% Coverage

R: Announcements should be made in the various communities so that people will be aware of the exercise and come out and test. (IDI-Parent with SCD Patient-Korania-07)

can meet and be educated on it.

I: What suggestions will you give to the nurses to help them do their work well?

R: if you inform the nurses well, they will in turn give out information to people.

<Files\\IDIs with SCD parents\\IDI-Parent with SCD patient-Navrongo-02> - § 6 references coded [11.13% Coverage]

Reference 1 - 2.21% Coverage

M: Good, what other suggestions would you make to improve this study or sickle cell testing exercise in your community?

R: I think it is very good so they should come and test their children to know their status but for me, myself I will not test but the children I will test them. I am saying this because when you know early it is better but for the reason is that when I was a child, I don’t fall sick so it means I don’t have sickle cell disease.

Reference 2 - 2.56% Coverage

M: What suggestions will you make about mothers and families accepting the testing and for their children to go and test.

R: I think if you know something it is the best way so if they go and test to confirm whether their children are positive or not, it will help them when the children are not having then it is good but if they have the parents or the family will be able to care for the children well and they will not be anemic. Since I have been going to the hospital the nurses help me a lot so they should also go.

Reference 3 - 1.92% Coverage

M: So, what did the nurse do to help you and you want to inform others.

R: I think they thought me, how to take care of the child may be the clothes, he will wear when the weather is cold, the food that we have to give to him, and maybe it is not all the medicines that I am supposed to give to the child. So, they too will advise you but if it is you, yourself you will not know what to do.

Reference 4 - 2.13% Coverage

M: Good, what suggestions about disclosing results of patients or participants?

R: There is no fear about it but the first time that they disclosed the result, I used to fear a lot but now I am used to it. So, now I know when someone is having sickle cell disease there is no fear because he/she is also normal like the way we are also living. It is only the frequent sickness that will bore the parents but there is no fear about it.

Reference 5 - 1.16% Coverage

R: Okay that is why I said to me there is no fear about disclosing the results whether the child will be positive or not and when the child is positive, they will put you on medications for the child to live normal without falling sick.

Reference 6 - 1.14% Coverage

R: They should all try their best and send their children to the clinics to test and know their sickle cell status and those who tested positive should pay attention to doctors’ advice so that their children will live a healthy life.

<Files\\IDIs with SCD parents\\IDI-Parent with SCD Patient-Nawognia-06> - § 3 references coded [6.96% Coverage]

Reference 1 - 1.70% Coverage

I: What do you suggest we do to make mothers and families accept the study in your community?

R: The parents always want to know the truth about everything and see the importance it will make. This will make it very easy for them to understand and when that is done, the parents will get their children involved. (IDI-Parent with SCD Patient-Nawognia-06)

Reference 2 - 2.39% Coverage

I: Okay. What about the use of community nurses, what can you say for them to do their work well?

R: They should control their anger because some parents do not have understanding and can easily pass a comment for the nurse to get angry, and when that happens, the nurse will not be able to work well. So, the nurses should control their anger and have patience with the parents and their children. This will help make the work successful.

Reference 3 - 2.87% Coverage

R: They should involve the leaders of the community to instruct their people to be part of the team to prove to the community that they know what you are about to do is good. When the people see their own people (leaders) among your team, they will believe because, if it is not good their leaders will not involve themselves to be part of the study team. (IDI-Parent with SCD Patient-Nawognia-06)

I: Okay.

R: Even the community members who will be part of the team will help explain to the study to the community members to understand.

<Files\\IDIs with SCD parents\\IDI-Parent with SCD Patient-Paga-05> - § 2 references coded [4.16% Coverage]

Reference 1 - 2.46% Coverage

I: What suggestion can you give to make parents and guardians accept the testing of their children that we intend to do?

R: I think that when you start announcing it on the various radio stations it will help, because some people have the disease but do not even know where to go for treatment. So if you announce it and tell us where to go and meet you for testing, and where to go for treatment, I think that would help. (IDI-Parent with SCD Patient-Paga-05)

Reference 2 - 1.70% Coverage

I: What concerns do you have when it comes to using the nurses for the testing, what advice do you have for them to make their work successful?

R: I think the nurses should always go with the machine (for outreaches) so that people will have trust in them, and it won’t be that they always come to talk and go.

<Files\\IDIs with SCD parents\\IDI-Parent with SCD-Pungu-04> - § 1 reference coded [1.88% Coverage]

Reference 1 - 1.88% Coverage

I: What do you have to add to make parents to accept for their children to take part in the testing when the project starts?

R: I first of all said prayer is the first thing. So, I will pray for the success of the project and for parents to understand the meaning of the project so that the project to help the children in living their lives. (IDI-Parent with SCD-Pungu-04)
