# Supplementary material for: Barriers and facilitators to community acceptability of integrating point-of-care testing to screen for sickle cell disease in children in primary healthcare settings in rural Upper East Region of Northern Ghana
Source: PLoS One. 2024 May 20;19(5):e0303520. doi: 10.1371/journal.pone.0303520 (PMC11104616; doi:10.1371/journal.pone.0303520)
Supplement: S2 Data — (ZIP) [file pone.0303520.s002.zip › S2_Data for health workers/A Views on SCD screening exercise.docx]

**Name:** Views on SCD screening exercise

<Files\\IDIs with com nurses\\IDI-26yr old community health nurse-Chiana-02> - § 2 references coded [4.78% Coverage]

Reference 1 - 1.85% Coverage

R: I think it will help a lot for us to detect our clients that are either AS, SS, we will be able to detect it early and refer such people for management because it is because we do not have the machine here, that is why we cannot tell whether we have them or we do not. So, I think the machine will help us to detect early and if there is the need for them to manage it, then we refer them for it to be managed

Reference 2 - 2.93% Coverage

R: what I will just have to add is that it is a good initiative, is very good. It will help us to detect… As I said, we do not know whether we have somebody like that coming to us for service or not because we do not have any machine to test them. So as this is coming, it will help, and my concern is if it comes, if it is starting then it should start. It should not be like a time where maybe the machines break down and they have to take like five months or so before they get it fixed. If they start the program, they should just make sure that everything is there for us to work with.

<Files\\IDIs with com nurses\\IDI-27yr old community health nurse-Chiana-01> - § 2 references coded [2.07% Coverage]

Reference 1 - 1.20% Coverage

R: This is a very good initiative, I think I am already in love with the idea though I have not seen the machine physically but it looks so simple to do as you have described. This is a very good initiative. It will help to intensify the education and since it is just here and there, you do the test and you are followed up, it is very good initiative.

Reference 2 - 0.87% Coverage

R: I think this is a very good initiative for the organization is a very good initiative and we have not yet started it but we are happy to participate when it comes. And we are saying on behalf of our mothers that we will do it and we will really be involved.

<Files\\IDIs with com nurses\\IDI-28yr old community health nurse-Wuru-06> - § 1 reference coded [3.06% Coverage]

Reference 1 - 3.06% Coverage

I: What do you think about this?

R: I think it is a good initiative if only you are going to do it as you say. It is going to help in a very good way. Because there are some that maybe they are even having the condition but do not know and sometimes you can even have somebody testing and the person is negative but maybe she is actually having it. It is a good initiative even though the mother may be negative but doing the test will come a long way to help.

<Files\\IDIs with com nurses\\IDI-29yr community health nurse-Biu-12> - § 2 references coded [2.55% Coverage]

Reference 1 - 0.74% Coverage

M: What do you think about us doing that?

R: Because you want to get children who are sickle cell positive. Bringing the machine here is purposely to get the children who are sickle cell positive.

Reference 2 - 1.81% Coverage

R: I just want to add a little to what we have discussed so I will just say we the community health nurses should agree to this program because it going to benefit the community and we are also part of the community like that so far as we are working in the community. When something good is coming out from the community our names too will also be mentioned so we should agree so that we will work hand-in-hand with the NHRC people for this activity to be carried out successfully.

<Files\\IDIs with com nurses\\IDI-29yr old community health nurse- Wuru-05> - § 1 reference coded [3.49% Coverage]

Reference 1 - 3.49% Coverage

I: This machine will be giving to the CHPS compounds and health facilities, to be used to test for sickle cell disease in the children. So, what do you think about us doing this?

R: I think it will help a lot because most children I will say die because of sickle cell and lack of ignorance and lack of machines like this. It is really causing harm, so if this machine comes, it will help us to be able to detect faster and refer for further treatment.

<Files\\IDIs with com nurses\\IDI-31yr old community health nurse-Biu-11> - § 2 references coded [3.99% Coverage]

Reference 1 - 3.18% Coverage

M: So, what do you think about us doing that?

R: It is a good initiative that you people are bringing and that way the parents will not avoid the health status of children concerning sickle cell or not. You know in a village like this the parents don’t care about the status of their children. So, when you tell them, they will say that they don’t have those kinds of sicknesses in this community but I think we have it. So, when this initiative comes like this and we can get some children who have sickle cell, it will help us the health workers to the kind of community and the children you are working with and the parents will also know the health status of their children.

Reference 2 - 0.81% Coverage

R: I will like to add that the project is a good initiative so when we start, we are begging that it should be continued and come to stay so that it will help the community.

<Files\\IDIs with com nurses\\IDI-33yr old community health nurse-Nabango-08> - § 1 reference coded [2.19% Coverage]

Reference 1 - 2.19% Coverage

I: What do you think about us doing that?

R: For parents to know their children’s sickle cell status or parents themselves to also know their sickle cell status. Because most of us, are living but don’t know our sickle cell status. So, the test, will help you to know your sickle cell status and decide what to do next and what not to do.

<Files\\IDIs with com nurses\\IDI33yr old Medical In-charge-Biu-09> - § 1 reference coded [0.53% Coverage]

Reference 1 - 0.53% Coverage

M: Good, what do you think about us doing that?

R: I think it is a good initiative and it will make our work very easy.

<Files\\IDIs with com nurses\\IDI-44yr old medical in-charge-Chaina-03> - § 2 references coded [3.28% Coverage]

Reference 1 - 1.73% Coverage

R: it is a good idea especially with the under 5s but I think that you are not training only the community health nurses for only the under 5s, so the maternity unit should also be involved. Is it that only one person is going to be trained to do both or you involve the maternity ward staff? This is because, once they are born and until they come for the first immunization, mostly they are with the midwife and after 24hrs, they are discharged home, then come back for the first immunization. So, are we involving the midwives or only the community health nurses or we will run it concurrently?

Reference 2 - 1.55% Coverage

R: I will say that it is a good thing to do for us to identify our under 5s with sickle cell which clinicians do not actually pay major attention to when we are in the consulting room. So I believe this will help us to be able to identify and manage them as early as possible so that we do not get our children passing away without knowing the diagnosis. Because mostly, when they die, they just burry them without autopsy to actually identify the cause, so this will actually help to reduce the number of under 5s deaths.

<Files\\IDIs with com nurses\\IDI-44yr old Medical in-charge-Wuru-07> - § 1 reference coded [5.40% Coverage]

Reference 1 - 5.40% Coverage

R: okay, then that will be fine for us. We can use this machine, it is okay.

I: So, what do you think about this project?

R: for me, I think it is welcome and I believe this will go a long way to help people in the community because most people are having diseases, especially sickle cell but do not know. I was once listening to a vice-chancellor who said he has stopped doing that work. He will counsel couples, go through the tests and realize they are sickle positive and that when they marry, children will come and some may suffer so he asks them to stop but they (couple) will not mind and complain that they have suffered together. So, the children will come out and be suffering. So, this test will help because when we get to know it early and we start to manage it very early, it will help because people think if you are sickle cell, you cannot cross 27 years. So, when we get to know early and start managing early, it will help save lives of the under 5s. it is a laudable idea, we will welcome it and we are all ready for it.

<Files\\IDIs with district and regional HWs\\IDI with public health nurse-02> - § 2 references coded [2.62% Coverage]

Reference 1 - 0.91% Coverage

M: Please what do you think about us doing that?

R: I think it is a good initiative that will go a long way to detect and prevent some deaths among children under five years old.

Reference 2 - 1.71% Coverage

M: Okay, is there anything else you will like to add that we have not already discussed?

R: I think we have discussed almost everything and this project coming in is a good initiative it will go a long way to create awareness of the disease condition and also alert us on the early detection of the disease if the machine is provided.

<Files\\IDIs with district and regional HWs\\IDI with Public health nurse-04> - § 1 reference coded [1.01% Coverage]

Reference 1 - 1.01% Coverage

M: What do you think about us doing that?

R: I think it will be very useful regarding the quality of services we provide at our health centers. I think if we have this machine in the health centers, it will provide better services to people very close to where they live.

<Files\\IDIs with district and regional HWs\\IDI-director of health services-01> - § 2 references coded [2.20% Coverage]

Reference 1 - 0.94% Coverage

R: I think it will be a very good intervention that will help the people and I will like to say that I am in support of the program.

Reference 2 - 1.26% Coverage

R: I think the questions have just covered all the areas but what

that we should make sure that the device is available in many facilities and it will help all the stakeholders that I mentioned early on.

R: I think it is a good initiative that will go a long way to detect and prevent some deaths among children under five years old.

R: I think it will be a very good intervention that will help the people and I will like to say that I am in support of the program.

<Files\\IDIs with district and regional HWs\\IDI-director of health services-03> - § 2 references coded [3.42% Coverage]

Reference 1 - 2.48% Coverage

M: What do you think about us doing that?

R: I can see it is a portable machine, depending on the power source, it can be used in all the facilities if it is not a machine that requires the use of national GRID. And I think it does not need any long training before you can be able to use it. So, I think doing this process is a good thing. As I said most facilities that conduct ANC, are a requirement for a sickle cell test. So probably, the study has to look beyond children, it should also look at the mothers of the children too.

Reference 2 - 0.94% Coverage

R: What I want to add is that, is a good study. When it comes, I will appreciate it. The only prayer is that this is a study, when it is to be implemented in full skill, we should give them a specialist.
