# Supplementary material for: Barriers and facilitators to community acceptability of integrating point-of-care testing to screen for sickle cell disease in children in primary healthcare settings in rural Upper East Region of Northern Ghana
Source: PLoS One. 2024 May 20;19(5):e0303520. doi: 10.1371/journal.pone.0303520 (PMC11104616; doi:10.1371/journal.pone.0303520)
Supplement: S2 Data — (ZIP) [file pone.0303520.s002.zip › S2_Data for health workers/B Using CHOs to screen children.docx]

**Name:** Views on Unsing of CHOs to screen children

<Files\\IDIs with com nurses\\IDI-26yr old community health nurse-Chiana-02> - § 1 reference coded [2.59% Coverage]

Reference 1 - 2.59% Coverage

R: Oh yes, it is possible.

I: How possible?

R: It is it possible because since it has to do with health and it has to do with saving people's lives, we are willing to do it, we will get a way to put it inside. Maybe when they come, we explain to them that there are a lot of things that come around and we have to add it, because we ourselves need to know our clients so we can help them. So, it is 100 percent possible because this one is not any extra work. It is just part of our work just that we do not have them. I think it is possible.

<Files\\IDIs with com nurses\\IDI-27yr old community health nurse-Chiana-01> - § 1 reference coded [1.82% Coverage]

Reference 1 - 1.82% Coverage

R: Yes, I think it is very possible because it is part of what we do already except for the technicality of the lab aspect. Apart from that, we attend to children so we will just inculcate it into one of our programs during the physical examination or education sessions time, we educate the mother and have it done. You said five minutes so the mothers will not be in a rush. Even if it is 20minutes, they will like to know because it is something that will help you to cater for your children so this is very good and it will be accepted everywhere.

<Files\\IDIs with com nurses\\IDI-28yr old community health nurse-Wuru-06> - § 1 reference coded [3.97% Coverage]

Reference 1 - 3.97% Coverage

R: Yeah, it is possible?

I: How possible will that be?

R: It is not going to be that easy, because definitely once it is coming with pricking the child and taking a sample from her, some of them will have questions as to why you are doing this and others will be resistant no matter what. We have different forms of human beings, so are very difficult so you have to convince them in a way to get them to agree before anything else can come. As you said, you are meeting the opinion leaders, meeting them to talk to them and they also communicate to the entire community will be a good thing.

<Files\\IDIs with com nurses\\IDI-29yr community health nurse-Biu-12> - § 3 references coded [4.67% Coverage]

Reference 1 - 1.75% Coverage

R: Yes, because most of the chef children when you get them in the child welfare clinic. They have been bringing a lot of children who are under five years to the child welfare clinic so when you have the opportunity to be at that place, you will get most of the children at that place to screen. If children are having sickle cell disease at that particular place, then if you test those children, you will be able to identify them and put them on medical treatment.

Reference 2 - 1.11% Coverage

M: Excellent, how will they do that, please.

R: I think if they know using it, they will be able to do that if they are taking through how to do it because I cannot just see the device and I will be able to use it, just like that. If I know using it then when it comes, I will be able to use it.

Reference 3 - 1.81% Coverage

R: I just want to add a little to what we have discussed so I will just say we the community health nurses should agree to this program because it going to benefit the community and we are also part of the community. When something good is coming out from the community our names too will also be mentioned so we should agree so that we will work hand-in-hand with the NHRC people for this activity to be carried out successfully.

<Files\\IDIs with com nurses\\IDI-29yr old community health nurse- Wuru-05> - § 2 references coded [6.42% Coverage]

Reference 1 - 2.93% Coverage

I: So how possible will nurses make this their routine duty to test for sickle cell disease in the facilities?

R: Okay. As a community health nurse, we work with children under six weeks to five years. So, with this machine, we can just put it at our place. When they come for weighing, we will just educate them on it and why we are doing the test. Whatever it is, we will refer.

Reference 2 - 3.49% Coverage

I: What are your concerns about nurses to appropriately test for sickle cell disease?

R: This one deals with blood and if we nurses will know how to handle the machine properly and how to manage it so that will not infect children and destroy the machine…. So the best way is for the nurses to use it because if we are educated, we will know how to use it and how to operate it in order not to affect the children or in order not to cause infections.

<Files\\IDIs with com nurses\\IDI-31yr old community health nurse-Biu-11> - § 1 reference coded [2.21% Coverage]

Reference 1 - 2.21% Coverage

R: Is possible that is if the children are not tested before.

M: Please, how will they do that?

R: If the device is not using electricity, they can carry it along when we are going for our routine visits such as outreaches, home visits, and even school health education. Even the machine can be in the health center and we will inform the children’s mothers to bring them to the facility for screening if we went to outreaches, home visits, and school health education.

<Files\\IDIs with com nurses\\IDI-33yr old community health nurse-Nabango-04> - § 1 reference coded [1.96% Coverage]

Reference 1 - 1.96% Coverage

R: Yeah, it is possible.

M: What will they do, can you explain how will they do that?

R: They have, now that you mentioned that the machine is movable, they do attributes and sometimes they do verbal and if they have one of these programs they can send the machine there, purposely they can organize a durbar just for the use of the machine to test children or any target group they identified that means the test we can do that.

<Files\\IDIs with com nurses\\IDI-33yr old community health nurse-Nabango-08> - § 1 reference coded [3.20% Coverage]

Reference 1 - 3.20% Coverage

I: Is it possible for the CHOs to routinely use the device to test children attending CHPS and immunization clinics for sickle cell disease?

R: Yes, very possible. Because they come every day and we meet children. We also go for a home visit, and out outreaches so with the children, we get them every day. And these children, we don’t know their sickle cell status. So, if we get the machine, we can be able to get their results instantly. And we will now see what help we can render to them.

<Files\\IDIs with com nurses\\IDI33yr old Medical In-charge-Biu-09> - § 1 reference coded [1.51% Coverage]

Reference 1 - 1.51% Coverage

R: Yes, is possible.

M: How will they do that please?

R: I think that first of all we have to get volunteers to announce to the community members where they will be going for their outreach. So, they will know what they are coming to do so that the community members will come out with their children for the exercise to be done successfully.

<Files\\IDIs with com nurses\\IDI-38yr old Medical In-charge-East-10> - § 2 references coded [5.91% Coverage]

Reference 1 - 4.75% Coverage

R: It will be the most appropriate intervention just like what you are saying mostly what we have is what we work with. We have RDT scripts so when they come whether complaints are geared towards malaria or not since that is the only thing, we have you will do the test to know whether the patients are suffering from malaria or not because you cannot just let the person come and go. If these sickle cell logistics were to be provided in addition, it is going to help just like you are saying mostly we know that sometimes we record deaths that are not even communicated to us because of some of these problems because they are just there and they don’t know what is wrong with the child and when they come, we too know that is not malaria but we don’t know what is happening to the child. So, we will refer them to the higher hospital but they even think of malaria and the result is positive so when you are referrer them most of them are not willing to go but if we can have the gaze machine first all. It will even ring in our head that sickle cell conditions are likely to be there and since the condition is likely to be there we will test to see and if the results come, we will give the appropriate education. So, apart from giving education to those who come and get the service, the presence of that machine is always a reminder to the health workers at CHOs or CHPS that this condition is there so always try to educate the people on it. So, I think it has a lot of benefits if only the machine will be given to the CHPS, CHOs, and immunization clinics.

Reference 2 - 1.15% Coverage

M: Please, I may ask how will they do that.

R: I know definitely when you have given them the machine, you will also train them so from training ones you know how to use it. At least I don’t think that we need lab technicians everywhere to do RDTs is the same thing. If they are also given the training and they know how to use it, I don’t think there is going to be a challenge.

<Files\\IDIs with com nurses\\IDI-44yr old medical in-charge-Chaina-03> - § 1 reference coded [1.38% Coverage]

Reference 1 - 1.38% Coverage

I: Alright, thank you. Please, is it possible for the community health nurses to routine do this in addition to their work?

R: It is possible.

I: How possible will that be?

R: if the machine is there and they are well educated and understand, it will be incorporated into their work schedule because with the nine months and one year immunizations, they do it. Looking at the machine, I think it should be easier to use because that was the first thing I wanted to ask.

<Files\\IDIs with com nurses\\IDI-44yr old Medical in-charge-Wuru-07> - § 1 reference coded [1.54% Coverage]

Reference 1 - 1.54% Coverage

I: so is it possible for community health nurses to routinely use this device to test for the disease?

R: yes, it can be done considering our line of work because we take care of them every day during our immunizations, so it will just be part of our work. It will even help boost our attendance

<Files\\IDIs with district and regional HWs\\IDI with public health nurse-02> - § 1 reference coded [1.72% Coverage]

Reference 1 - 1.72% Coverage

M: Excellent, is it possible for CHOs to routinely use the device to test children attending CHPs and immunization clinics for sickle cell disease?

R: Yes, is it and that will be based on the training needed if the machine is provided, they need to train on how to use the device for testing effectively so that we will get good results.

<Files\\IDIs with district and regional HWs\\IDI with Public health nurse-04> - § 1 reference coded [2.11% Coverage]

Reference 1 - 2.11% Coverage

M: Is it possible for the CHOs to routinely use to test children attending CHPS and immunization clinics for sickle cell disease?

R: I just looked at the process and I think if they are given the capacity, they can do it. Because some parts of the machine look like the IGTs we already use in the system. Perhaps some parts of it that will be a challenge. But if we are to take the blood sample, we are already conversant with that. They have taken samples several times so, I think they can do it too is only a few things that they may have challenges in doing it.

<Files\\IDIs with district and regional HWs\\IDI-director of health services-01> - § 2 references coded [4.67% Coverage]

Reference 1 - 3.26% Coverage

M: Good, is it possible for the CHOs to routinely use the device to test children attending CHPs and immunization clinics for sickle cell disease?

R: It is very-very possible because of sickle cell prevalence we have in this district or region as we compare to malaria. So, I am sure it will not be a big burden at all. The only challenge may be that I don’t know the way the machine will work and whether it will use electricity or not because some of the facilities don’t have electricity. So, that will be a challenge, I am sure if they get the machine and get the signs and symptoms of sickle cell, they should be able to test and get the results.

Reference 2 - 1.41% Coverage

M: How will they do that, please.

R: So, if the device is available and you train them on how to test, when they get patients who are probably always showing the signs and symptoms of the sickle cell then you can suspect that patient and do a test to confirm positive or negative.

<Files\\IDIs with district and regional HWs\\IDI-director of health services-03> - § 2 references coded [3.50% Coverage]

Reference 1 - 2.39% Coverage

M: Is it possible for the CHOs to test children attending CHPS and immunization clinics for sickle cell disease?

R: Well, CHPS is for community health nurses CHOs and as part of their mandate at the CHPS compound, they are supposed to attend to every person that comes. And early detection, if they are trained and can use the machine, they can identify why the person is sick. So, that becomes a quality indicator at the CHPS level if they can identify sickle cell disease. Cost management will be done elsewhere.

Reference 2 - 1.11% Coverage

M: Pease, how will they be able to use the machine and do that?

R: Once they are trained. This requires some level of training I don’t how long it will take but, it shouldn’t take much time to orient someone to be able to use the machine.
