# Supplementary material for: Barriers and facilitators to community acceptability of integrating point-of-care testing to screen for sickle cell disease in children in primary healthcare settings in rural Upper East Region of Northern Ghana
Source: PLoS One. 2024 May 20;19(5):e0303520. doi: 10.1371/journal.pone.0303520 (PMC11104616; doi:10.1371/journal.pone.0303520)
Supplement: S2 Data — (ZIP) [file pone.0303520.s002.zip › S2_Data for health workers/C Factors affecting acceptability.docx]

**Name:** E Factors affecting acceptability

<Files\\IDIs with com nurses\\IDI-26yr old community health nurse-Chiana-02> - § 2 references coded [3.01% Coverage]

Reference 1 - 2.01% Coverage

R: I think sometimes the community members are difficult and some of them even though you are helping them, they will not patronise the exercise but when they get to know that you are not giving me money, you would not do this and this, they turn to withdraw even though what you are doing is to help them. So, if you implement it this way and they have to go to the hospitals to pay bills and do all those things, it may affect the program.

Reference 2 - 1.00% Coverage

R: I think so, the rest will be

Also, if we the health workers turn to disclose their information to people, they will not trust us again and it will prevent others from accepting what we are giving to them.

<Files\\IDIs with com nurses\\IDI-27yr old community health nurse-Chiana-01> - § 1 reference coded [2.53% Coverage]

Reference 1 - 2.53% Coverage

I: Do you think there are factors relating to our culture or religion that can prevent people from accepting this sickle cell testing of children?

R: I wouldn't really know of other doctrines. But with the doctrines practised here, there is no doctrine that would really stop us from checking children for their sickle cell. I don't know about other communities, but in this community, with my knowledge, there is no doctrine, whether the African traditional religion, Christianity, Islam, they do not mind. That is what is mainly here. The other ones, I've not really seen, the churches, I have not really seen any church that is against taking of blood sample and conducting a test, no. So, for all the doctrines practised here, they will really accept it.

<Files\\IDIs with com nurses\\IDI-28yr old community health nurse-Wuru-06> - § 1 reference coded [3.36% Coverage]

Reference 1 - 3.36% Coverage

I: Are there factors that are likely to affect the acceptance of this study in this community? Religiously, culturally, will there be challenges?

R: For the acceptance, yes, culturally, there are some who will deny it and say such conditions do not exist and that is when they will go in for the local treatments. And religiously, there are some people who are too religious. No matter what you say, they will say that God has said that my child will be healed even without medication. So, it is possible.

<Files\\IDIs with com nurses\\IDI-29yr community health nurse-Biu-12> - § 1 reference coded [1.74% Coverage]

Reference 1 - 1.74% Coverage

R: Some of the religions don’t believe those testing things even in our local family, you will go and they will say that it is not working. Let’s do it locally so some of them when you even want to do the test, might not even agree because their belief is just that the ancestors will heal the child or the local herbs can treat the child. Those are the things that will affect the acceptance of the testing and the acceptance will not be massive in the community.

<Files\\IDIs with com nurses\\IDI-29yr old community health nurse- Wuru-05> - § 1 reference coded [3.60% Coverage]

Reference 1 - 3.60% Coverage

I: What general factors do you think is likely to affect the acceptance of this device, in regards to religious beliefs and their cultural beliefs?

R: Well, for me, there will not be any challenge but if only it is going to be having any cost, that is what will be a problem. Because here, if we are going to place a cost on the testing and all those things, it will discourage some people from taking part in the exercise or it is not covered by insurance or so and they have to pay for it, I do not think they will encourage it.

<Files\\IDIs with com nurses\\IDI-31yr old community health nurse-Biu-11> - § 4 references coded [4.53% Coverage]

Reference 1 - 0.73% Coverage

R: If there is payment for testing and if there is a long time waiting for testing and that of results. If it falls on a market day, you will not get them.

Reference 2 - 1.28% Coverage

M: Okay, so what are the religious factors that can affect the acceptance of testing for sickle cell disease in this community?

R: Some people will say my grandparents said they should not use their blood for testing or at our house they don’t take our blood to do testing. Some churches too are there; they don’t allow people to take their blood samples for testing.

Reference 4 - 1.91% Coverage

M: You know religious beliefs can be traditional, Christianity, Islam, and so on but now cultural beliefs. Our way of life can affect the acceptance of sickle cell testing in this community.

R: In my village here, I have never seen anybody who said they don’t take their child’s sample or screen the child, I don’t think there are cultural beliefs that can affect the testing of sickle cell in my community.

<Files\\IDIs with com nurses\\IDI-33yr old community health nurse-Nabango-04> - § 5 references coded [6.06% Coverage]

Reference 1 - 1.49% Coverage

M: Okey excellent, generally what factors do you think are likely to affect the acceptance of sickle cell disease testing exercise in this community?

R: I will only mention one and that one is when the testing has to do with the payment of money. That will be the major factor that will affect the acceptance of the testing.

Reference 2 - 2.00% Coverage

M: So, what will be the religious factors that will affect the acceptance of the test?

R: Mostly, we don’t have that kind of resistance often, except that those who have boils on their bodies and they don’t accept vaccination. They don’t agree so maybe those and then but apart from that, I haven’t seen any religious resistance. If somebody has boils, they don’t accept injections and I don’t know why they don’t but that is what I know.

Reference 3 - 0.64% Coverage

M: What about cultural beliefs?

R: That one I’m not too sure, though it is there I haven’t met one I can’t say it wouldn’t be there, we may.

Reference 4 - 1.58% Coverage

M: So, what suggestions to overcome the money factor you early mentioned?

R: I don’t know maybe after giving the items out is it going to be a continuous process like when the things finish you supply them again and the maintenance. If it is going to be the facility that is going to be doing the maintenance, then it is going to be a challenge.

Reference 5 - 0.36% Coverage

R: I suggest that they should do it like the malaria test without taking money.

<Files\\IDIs with com nurses\\IDI-33yr old community health nurse-Nabango-08> - § 1 reference coded [10.34% Coverage]

Reference 1 - 10.34% Coverage

I: Generally, what factors do you think are likely to affect the acceptance of sickle cell disease testing exercises in this community?

R: If the assemblymen, the chief, the youth organizers, and the leaders of the community people are not involved, the community people will not accept it. That is one thing we observed here. Just like the covid vaccine we were giving, we started with the chiefs, so at least with them, then we the staff, if it is school, we start with the teachers. Then they now believe that they can also take it. So, it is the same way, if we involve them. Maybe the day for the durbar or whatever we are to give the information, if they see them coming or they are being, seated, it will encourage others to come to join and it will be successful.

I: What are the religious beliefs factors that are likely to affect the testing exercise?

R: The religious beliefs, when it comes to church, the pastors have heard about it and they even know a bit about it. So, if you involve the pastor, and maybe in the church service, the pastor himself is involved in the talking, at least they will know that there is time for everything, there is time for God to deliver them and some of them unless the drugs that can heal them.

I: What about the cultural beliefs?

R: The cultural beliefs too, they have to get to those who are into herbs and the traditional leaders and involve them so they will also share their ideas or perception about that then you will add the clinical part of the medical part. With that, they will know that they have to do this, they have to do that.

<Files\\IDIs with com nurses\\IDI33yr old Medical In-charge-Biu-09> - § 4 references coded [8.72% Coverage]

Reference 1 - 4.52% Coverage

R: The challenges that they will face will be clients because some of them might not agree to do the test. Another challenge will be the maintenance that I made mentioned earlier and for now, the usage. The one who will be very competent to use it because it is a new machine that has just come and they might not have enough knowledge to use it.

M: So, can you give us ways to address these challenges that you mention?

R: First of all, if we can get personnel from the NHRC who know how to use the device to be able to take them through and then more to the point the maintenance if there is a way that if the machine gets a fault or had any issues so that they can get somebody to work on it quickly. So, the device can continue working for the success of the project, and then we have to organize a durbar so that the information will get to all community members, so, that they will be aware that this is what is going on and they can come out with their children for the exercise to be carried out based on their will.

Reference 2 - 1.39% Coverage

R: This community since I came here is going two years now and what I have observed is that the community people are always happy when initiatives like this come up. They are always willing to come out and support and partake in any issue that is concerning their health so I think that the acceptance will be good.

Reference 3 - 1.79% Coverage

M: What are the religious beliefs that can affect the acceptance of the sickle cell testing exercise?

R: They might believe that sickle cell disease is not a medical condition and is treated traditionally. So, that belief combined with some of them saying that the woman went out to have affair with someone outside, is why the child is suffering like that, I think that one is also another major belief.

Reference 4 - 1.03% Coverage

M: What about cultural beliefs that can affect the acceptance of the sickle cell testing exercise?

R: Some people traditionally don’t allow their family members to be tested in the hospital and they always believe in the herbalists.

<Files\\IDIs with com nurses\\IDI-38yr old Medical In-charge-East-10> - § 3 references coded [5.02% Coverage]

Reference 1 - 1.23% Coverage

R: So, I don’t think that people will refuse to do the testing if we factor it into our routine services but if we want it to look like a special thing that is where the acceptability will be difficult. If we are going to make it look like we are to test and see whether the person has sickle cell disease or not by communicating well and effective networking then I don’t think they will be any challenge.

Reference 2 - 3.09% Coverage

M: Please, can you give some religious beliefs that will affect the acceptance of sickle cell disease testing in this community?

R: For now, I think it is just like what I have said that we don’t let it look like it is especially from what we are doing but it is also going to be part of our routine service. Knowing the status of somebody suppose to be a routine service and though you are not going to deceive them, most people refuse to do HIV/AIDS tests and counseling because they have not been made to understand that it is supposed to be part of the service. That is why the fear is there but if the service is holistic and everything is there to do the test and if any patient comes, he/she should also go through all the tests. So, I think it will depend on how you people want it and if you want to factor it as part of the routine service it will help but if you want it to be like a special thing that is where you can counsel thousand people but at the end. You will not get any person to go for the test.

Reference 3 - 0.69% Coverage

M: Please, what about cultural beliefs too?

R: I don’t think cultural beliefs can affect you because you cannot go to the hospital and tell the health workers that you need this and you don’t need this and they will accept you.

<Files\\IDIs with com nurses\\IDI-44yr old medical in-charge-Chaina-03> - § 2 references coded [2.53% Coverage]

Reference 1 - 1.98% Coverage

I: Alright. Please are there any cultural issues or religious issues you think can affect the acceptance of this new development or project, for the parents to allow their children to be tested? Are there any factors that can affect it?

R: For working here for almost close to five years, I have not come across any. Maybe it will be the Jehovah Witness who will say that no blood transfusion and all those things but taking a blood sample for testing, I have not encountered that. As for blood transfusion, that is where we will have challenges where they say that when they donate, they cannot do their work. That is the only thing we know of but to pick blood for testing, no.

Reference 2 - 0.55% Coverage

I: So with the Jehovah Witness case, how will that be addressed?

R: We have not encountered some because they agree to prick for testing but it is the transfusion they have problem with.

<Files\\IDIs with com nurses\\IDI-44yr old Medical in-charge-Wuru-07> - § 1 reference coded [3.17% Coverage]

Reference 1 - 3.17% Coverage

R: So far, we have done a lot of projects in this area, from Korania to Pindaa. We have never had a situation militating progress of any project, be it religious beliefs, cultural beliefs or anything. This program will not be an exception there will not be any bridge that will...there is nothing like that here and we have never had something like that. We have taken peoples’ blood to do other projects which are not even from your place but there are no restrictions as far as I am concerned. So, I do not think this program will have impediments that may go against the implementation, it will sail through like others.

<Files\\IDIs with district and regional HWs\\IDI with public health nurse-02> - § 3 references coded [4.86% Coverage]

Reference 1 - 1.81% Coverage

M: Generally, what factors do you think are likely to affect the acceptance of sickle cell disease testing exercises in this community?

R: I think that will be the attitude of some staff. So, I think how the staff is going to work with the parents and how they will talk to them may affect interest of some community members.

Reference 2 - 1.97% Coverage

M: What religious beliefs do you think are likely to affect the acceptance of this sickle cell disease in this region/district?

R: But this I think the municipal we don’t have many religious beliefs like that sort but it is only a few that do not even allow their children to come for health facility for immunization with they believe that their gods will take care of their children.

Reference 3 - 1.09% Coverage

M: What about the cultural beliefs?

R: Actually, the testing I don’t think will be a challenge in the communities once we create awareness. I think they will accept it and they will even appreciate it very well.

<Files\\IDIs with district and regional HWs\\IDI with Public health nurse-04> - § 2 references coded [2.88% Coverage]

Reference 1 - 1.69% Coverage

R: I think literacy will be one. Those that are not educated probably will have those little difficulties in accepting the test compared to compared who are educated. People who have previous knowledge about the condition will be more willing to accept it than those who don’t even know about it. And mostly, we subscribe to traditional medicine compared to compare doctors so, so that could also be a factor that can affect the acceptance of the testing.

Reference 2 - 1.19% Coverage

M: Religious beliefs?

R: Mostly in the traditional homes if the child is sick, they will make inquiries from the soothsayers but, I am not sure that the soothsayer will say it is a sickle cell. So, such a person when they bring to the facility, we test and explain the condition of the child to them and they believe it.

<Files\\IDIs with district and regional HWs\\IDI-director of health services-01> - § 2 references coded [4.18% Coverage]

Reference 1 - 1.03% Coverage

M: Good boss, generally, what factors do you think are likely to affect the acceptance of sickle cell disease testing exercise in this community?

R: I don’t see any challenges associated with that, please.

Reference 2 - 3.15% Coverage

M: What are the religious believes or cultural beliefs that you think are likely to affect the acceptance of sickle cell disease testing in these communities?

R: For testing, I am not sure, okay because it is an intervention, you have to take the blood of the person. When you are doing community exercise as when the clients come to the facility what I know they are always willing to go through to do the testing. Most of the people when they are sick and go to hospital, they want their blood to be tested so, for children who have signs and symptoms, I am very convinced that parent will corporate for the testing to be done.

<Files\\IDIs with district and regional HWs\\IDI-director of health services-03> - § 3 references coded [8.53% Coverage]

Reference 1 - 2.05% Coverage

M: Generally, what factors do you think are likely to affect the acceptance of sickle cell disease testing exercises in this community?

R: Depending on how it all starts, the approach, at the pre-implementation stage, People need to be given the right orientation to accept it. And it also depends on how much the charge will be if there is going to be a monitory thing in it. Some may not agree to pay for the testing, if it is free then fine.

Reference 2 - 2.72% Coverage

M: What are the religious beliefs that are likely to affect the acceptance of the testing?

R: I don’t know any and I have never heard of any.

M: What about the cultural beliefs?

R: That one too, I don’t have an idea. But sorry, it has to do with the taking of blood? I could remember when we were doing the covid vaccination, they went to one mosque and they refused. It was during the time of fasting and cost, I know children don’t fast. But it was adults who were fasting, they say if you spill blood, it among to breaking of your fast. That’s the only thing I can think of for now.

Reference 3 - 3.75% Coverage

M: Cultural beliefs?

R: I have not heard of it yet but, I know some people do not accept orthodox treatments of cases. That is all I know but I have never heard of it here in the Kassena Nankana west since I came. I know we have been vaccinating since I came here, it used to be those days when you ask people to go for vaccination and they tell you my children don’t take vaccines. Even for those who go to the traditional healers, we have been sending our concerns to them that when they know they cannot treat it, they should refer to us and some have been referring. There is this man in Kayoro, during the covid time he referred one of the cases to the health center and subsequently, the person came here. So, some of the traditional treatment people refer cases to us depending on your linkage to them.
