# Supplementary material for: Barriers and facilitators to community acceptability of integrating point-of-care testing to screen for sickle cell disease in children in primary healthcare settings in rural Upper East Region of Northern Ghana
Source: PLoS One. 2024 May 20;19(5):e0303520. doi: 10.1371/journal.pone.0303520 (PMC11104616; doi:10.1371/journal.pone.0303520)
Supplement: S2 Data — (ZIP) [file pone.0303520.s002.zip › S2_Data for health workers/D Suggestions to improve acceptability.docx]

**Name:** Solutions to address these factors

<Files\\IDIs with com nurses\\IDI-26yr old community health nurse-Chiana-02> - § 1 reference coded [2.33% Coverage]

Reference 1 - 2.33% Coverage

R: if we say we are going to do something, then the things we need to do the work should be there. The device should be functioning all the time, it should not be a case where maybe a mother would come and we will be like the thing has spoiled or today we cannot do it. That way, we ourselves will feel someway and it will be like we are not even serious. So, I think the things we will need to work with should always be there, that way it will motivate us to work.

<Files\\IDIs with com nurses\\IDI-29yr community health nurse-Biu-12> - § 1 reference coded [2.22% Coverage]

Reference 1 - 2.22% Coverage

R: Initially, I spoke about education and health talks, I think those are the factors when we base on and educate them very well to understand the need for the exercise they will embrace it and come for the screening for them to know that those religious beliefs are not relevant again because though we know God can do things but that is why science is also there. God is healing but the healing normally passes through someone and touches you so the scientist in the hospital can treat us based on the wisdom they got from God. So, I think health education, home visitations, and so on about the testing, and community members need to know their children’s sickle cell status.

<Files\\IDIs with com nurses\\IDI-31yr old community health nurse-Biu-11> - § 1 reference coded [2.11% Coverage]

Reference 1 - 2.11% Coverage

M: Okay, what can we do to overcome these factors or challenges mentioned above?

R: Health education for them to know that taking the blood sample is not that we are going to use it for something else but it is just for the testing to see whether the child is having sickle cell or not and after that nobody need their blood. So, we will put the child on medication for the management of the disease which will improve the child’s living conditions.

<Files\\IDIs with com nurses\\IDI-33yr old community health nurse-Nabango-04> - § 2 references coded [1.94% Coverage]

Reference 1 - 1.58% Coverage

M: So, what suggestions to overcome the money factor you early mentioned?

R: I don’t know maybe after giving the items out is it going to be a continuous process like when the things finish you supply them again and the maintenance. If it is going to be the facility that is going to be doing the maintenance, then it is going to be a challenge.

Reference 2 - 0.36% Coverage

R: I suggest that they should do it like the malaria test without taking money.

<Files\\IDIs with com nurses\\IDI-33yr old community health nurse-Nabango-08> - § 1 reference coded [2.37% Coverage]

Reference 1 - 2.37% Coverage

I: What suggestion to overcome these factors or challenges mentioned above?

R: As I said earlier, it means

We have to involve everybody from the traditional leaders, the pastors, the imams, the youth organizers, the chiefs, the elders, and the youth. We have to involve all of them in the program they are leaders. If we can get them, then the information will get to them well.

<Files\\IDIs with com nurses\\IDI33yr old Medical In-charge-Biu-09> - § 2 references coded [6.43% Coverage]

Reference 1 - 4.52% Coverage

R: The challenges that they will face will be clients because some of them might not agree to do the test. Another challenge will be the maintenance that I made mentioned earlier and for now, the usage. The one who will be very competent to use it because it is a new machine that has just come and they might not have enough knowledge to use it.

M: So, can you give us ways to address these challenges that you mention?

R: First of all, if we can get personnel from the NHRC who know how to use the device to be able to take them through and then more to the point the maintenance if there is a way that if the machine gets a fault or had any issues so that they can get somebody to work on it quickly. So, the device can continue working for the success of the project, and then we have to organize a durbar so that the information will get to all community members, so, that they will be aware that this is what is going on and they can come out with their children for the exercise to be carried out based on their will.

Reference 2 - 1.91% Coverage

M: Please what again, okay suggestion to overcome these factors or challenges mentioned above?

R: I think basically, education will do by organizing durbars to talk about it and then letting them understand that the sickle cell disease is not traditional herbs that can treat it. I think if they get that understanding, it will help of the commission of the project, they will appreciate it

<Files\\IDIs with com nurses\\IDI-44yr old medical in-charge-Chaina-03> - § 1 reference coded [0.55% Coverage]

Reference 1 - 0.55% Coverage

I: So, with the Jehovah Witness case, how will that be addressed?

R: We have not encountered some because they agree to prick for testing but it is the transfusion they have problem with.

<Files\\IDIs with district and regional HWs\\IDI with Public health nurse-04> - § 1 reference coded [3.17% Coverage]

Reference 1 - 3.17% Coverage

M: Your suggestions to overcome these factors you or the challenges you mentioned?

R: I think we should be doing the sensitization so that everyone becomes aware of it in the community. And also, the religious angle, we can remedy it to a lot extent because once people are aware and the discussions things will tell them to be manifesting with children then, they are likely to also believe us. Then the second thing is having special attention for these traditional leaders and the soothsayers so that they know that they are also around to diagnose some of these conditions and sometimes they should refer to the facility so that we can diagnose them. You should educate them well enough so that if they believe what we say, I think is just like a collaboration. So, I think if you can do this, some of them reserve their comments on that part of it.

<Files\\IDIs with district and regional HWs\\IDI-director of health services-03> - § 1 reference coded [2.24% Coverage]

Reference 1 - 2.24% Coverage

M: What are your suggestions to overcome these factors or challenges you talked about?

R: I think there should be proper engagement with the identified groups. You talk to them. One is religion, it is research that shows that when you spill blood it doesn’t mean you have broken your fast. You can use that one to change your mindset. So, we have to do a lot of research into those things so that we can use that as a means of communication with the groups that have that problem.
